# Peptide-based semiconducting polymer nanoparticles for osteosarcoma-targeted NIR-II fluorescence/NIR-I photoacoustic dual-model imaging and photothermal/photodynamic therapies

**DOI:** 10.1186/s12951-022-01249-4

**Published:** 2022-01-21

**Authors:** Ying Yuan, Shanchao Diao, Xiaoyue Ni, Dong Zhang, Wanrong Yi, Chao Jian, Xiang Hu, Daifeng Li, Aixi Yu, Wen Zhou, Quli Fan

**Affiliations:** 1 Department of Orthopedics Trauma and Microsurgery, Zhongnan Hospital of Wuhan University, Wuhan, 430071 China; 2 State Key Laboratory of Organic Electronics and Information Displays & Institute of Advanced Materials (IAM), Nanjing University of Posts & Telecommunications, Nanjing, 210023 China; 3 Department of Magnetic Resonance Imaging, Henan Key Laboratory of Functional Magnetic Resonance Imaging and Molecular Imaging, The First Affiliated Hospital of Zhengzhou University, Zhengzhou, 450052 China

**Keywords:** Dual-modal imaging, Photothermal therapy, Photodynamic therapy, Osteosarcoma-targeted

## Abstract

**Background:**

The overall survival rate of osteosarcoma (OS) patients has not been improved for 30 years, and the diagnosis and treatment of OS is still a critical issue. To improve OS treatment and prognosis, novel kinds of theranostic modalities are required. Molecular optical imaging and phototherapy, including photothermal therapy (PTT) and photodynamic therapy (PDT), are promising strategies for cancer theranostics that exhibit high imaging sensitivity as well as favorable therapeutic efficacy with minimal side effect. In this study, semiconducting polymer nanoparticles (SPN-PT) for OS-targeted PTT/PDT are designed and prepared, using a semiconducting polymer (PCPDTBT), providing fluorescent emission in the second near-infrared window (NIR-II, 1000 - 1700 nm) and photoacoustic (PA) signal in the first near-infrared window (NIR-I, 650 - 900 nm), served as the photosensitizer, and a polyethylene glycolylated (PEGylated) peptide PT, providing targeting ability to OS.

**Results:**

The results showed that SPN-PT nanoparticles significantly accelerated OS-specific cellular uptake and enhanced therapeutic efficiency of PTT and PDT effects in OS cell lines and xenograft mouse models. SPN-PT carried out significant anti-tumor activities against OS both in vitro and in vivo.

**Conclusions:**

Peptide-based semiconducting polymer nanoparticles permit efficient NIR-II fluorescence/NIR-I PA dual-modal imaging and targeted PTT/PDT for OS.

**Graphic Abstract:**

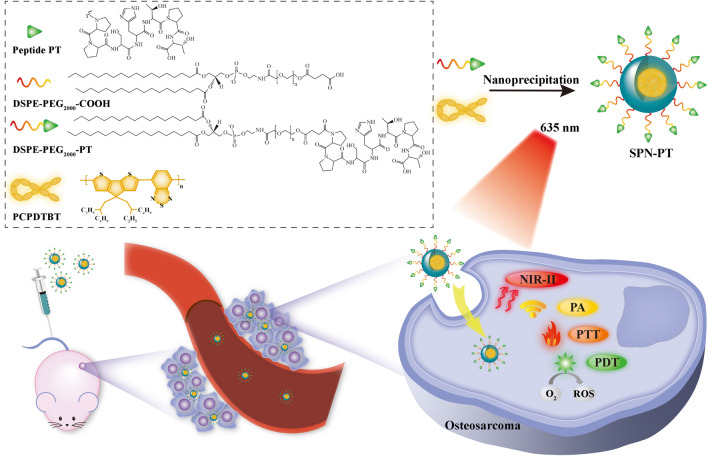

**Supplementary Information:**

The online version contains supplementary material available at 10.1186/s12951-022-01249-4.

## Introduction

Osteosarcoma (OS) is the most common malignant and aggressive primary bone tumor that affects children and adolescents, which is not only locally destructive but also highly metastatic [1, 2]. Currently, combined regimens of poly-chemotherapy and surgical removal of the primary tumor along with all clinically evident metastatic disease remain major management strategies for OS patients [3–5]. However, characteristics like invasive growth and close adhesion to surrounding tissues of OS make it difficult to completely eliminate the tumor cells during surgery and prevent cancer recurrence in clinic [6, 7]. Simultaneously, the extensive presence of dormant micrometastatic lesions in OS patients at the time of diagnosis, and the minimal residual recurrent diseases following standard therapeutics cause an even worse survival condition in OS patients [8]. Innovative diagnostic techniques with better sensitivity to OS are needed for early diagnosis and therapeutic guidance and monitoring, in allusion to the minimal lesions. Attempts have been made to fabricate novel probes or nanocarriers for more sensitive diagnosis or efficient drug delivery for OS [9–11]. Nevertheless, no molecular signatures, which would make biologically targeted therapy and diagnosis promising, have been identified as robust prognostic indicators of overall survival or response to chemotherapy in OS [12]. Therefore, a theranostic method equipped with good sensitivity, high targeting ability and therapeutic efficiency for OS is urgently need.

In recent years, molecular optical imaging with multi-functional biomaterials enables early non-invasive diagnosis, real-time monitoring, and accurate surgical guiding with high sensitivity [13]. Among numerous imaging modalities, fluorescence imaging in the first and the second near-infrared region (NIR-I, 650–900 nm; NIR-II, 1000 - 1700 nm) and photoacoustic (PA) imaging have been widely utilized benefited from their superior penetration depth in vivo [14–16], which are, therefore, deep tissue imaging applicable and efficient. Complementary to conventional optical imaging technologies, dual-modal imaging combining PA and NIR-II fluorescence is characterized with not only high spatial resolution and outstanding anatomical delineating capability of PA imaging [17], but also with significantly reduced photon scattering and minimal tissue-derived autofluorescence of NIR-II fluorescence imaging [14]. Besides serving as excitation of optical imaging, light can trigger phototherapies including photothermal therapy (PTT) and photodynamic therapy (PDT), emerged with excellent spatial specificity and noninvasiveness compared with traditional chemotherapies and radiotherapies [18, 19]. The so-called phototheranostics which combines phototherapies and optical imaging not only permits precise administration of phototherapies under imaging guidance, but also realizes real-time therapeutic effects monitoring, thus reduces side effects and prevents recurrence. Therefore, development of biomaterials for NIR-II fluorescence/PA imaging-based phototheranostic systems is highly demanded.

To date, however, the uptake of biomaterials into tumor tissues and cells is mostly the controversial enhanced permeability and retention (EPR) [20–22]. It has been recommended that active transcytosis techniques be developed for delivery efficiency enhancement [21, 23], including utilization of tumor-penetrating peptides [24] and design of polymer conjugates [25]. Providing with high binding affinity and specificity to certain extracellular ligands, targeting peptides possess advantages including convenient synthesis and easy chemical modification, good histocompatibility, low immunogenicity, favorable pharmacokinetics, and acceptable in vivo stability and integrity [26, 27], and have emerged as an alternative to molecular targeting ligands [28]. Among these, peptide PT (PPSHTPT) has been constructed to mimic natural protein osteocalcin property in vivo and has shown its targeted binding to osteosarcoma in vitro and in vivo [29–31].

Herein, we report an oligopeptide PT based semiconducting polymer nanoparticles (SPN-PT), which shows OS targeting ability, for NIR-II fluorescence and NIR-I PA dual-modal imaging guided PTT and PDT therapies. Semiconducting polymer nanoparticles (SPNs) represent an emerging class of organic photonic agents fabricated by optically active semiconducting polymers [32], which have been tremendously explored for the applications such as fluorescence imaging [33], PA imaging [34], PTT [35] and PDT [36]. SPN-PT is formulated by nanoprecipitation method using a semiconducting polymer (PCPDTBT) encapsuled with polyethylene glycolylated (PEGylated) PT peptide to improve water solubility. With PT peptide, the SPNs can be internalized into OS cells in an active fashion, showing a faster uptake and good selectivity, permitting accurate early diagnosis for OS by NIR-II fluorescence and PA signals, providing efficient PTT and PDT in vitro and in vivo. In a word, an OS-targeted nanoplatform with dual model imaging ability and multi-phototherapy effects is introduced, which enables OS early diagnosis with its high sensitivity and selectivity, and efficient treatments for OS with its phototherapies as well as targeting abilities.

## Results and discussion

### Construction and characterization of nanoprobe

**Scheme 1:**
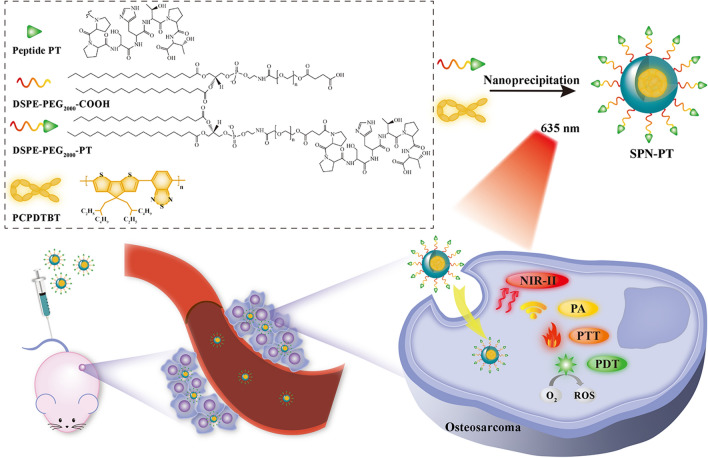
Schematic illustration of the preparation process of SPN-PT and NIR-II fluorescence/NIR-I PA imaging and targeted PTT/PDT against osteosarcoma

To prepare SPN-PT, amphiphilic copolymer PEG-PT was first synthesized by conjugating peptide PT onto the carboxyl group of DSPE-PEG_2000_-COOH via amidation reaction (Scheme 1). Another copolymer PEG-SP was synthesized by changing PT into peptide SP via similar procedure. The structure of PEG-PT was characterized by proton nuclear magnetic resonance (^1^ H HMR) and matrix assisted laser desorption ionization-time of flight mass spectrometry (MALDI-TOF-MS) (Additional file 1: Figure S1–S5). SPN-PT and its counterpart SPN-SP were then prepared via a nanoprecipitation method which used PEG-PT or PEG-SP, respectively, to encapsulate hydrophobic semiconducting polymer PCPDTBT.

**Fig. 1:**
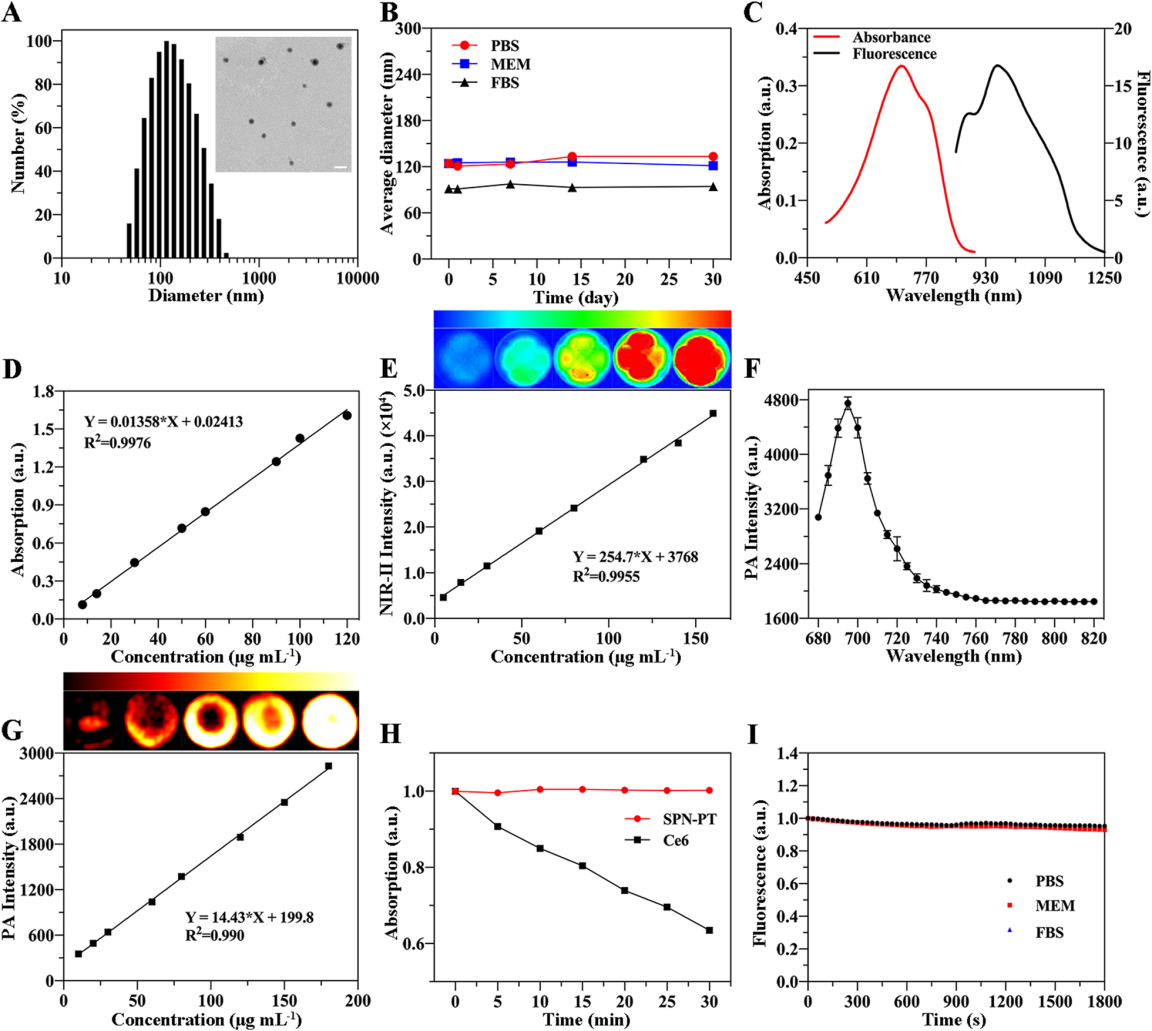
Characterization of SPN-PT photophysical properties. **A** Representative TEM image and DLS analysis of SPN-PT. Scale bar = 500 nm. **B** Stability of averaged diameters of SPN-PT in PBS, MEM and FBS during the storage for 30 days. **C** Absorption spectra (red) and corresponding fluorescence emission spectra (black) of SPN-PT. **D** The UV absorption standard curve of SPN-PT aqueous solution at 700 nm. **E** Representative NIR-II fluorescence images (upper) and NIR-II fluorescence intensities with the concentrations of SPN-PT under 808 nm excitation with a 980 nm filter. **F** PA imaging spectra (680 to 820 nm) of SPN-PT (100 µg mL^−1^). **G** Representative PA images (upper) and the fitting curve of PA intensity of SPN-PT at 695 nm against concentrations (lower). **H** Normalized UV absorption stability of SPN-PT and Ce6 at 700 nm and 640 nm, respectively. The solutions were irradiated by a 635 nm laser at 0.75 W cm^−2^. **I** Photostability monitored of SPN-PT in PBS, MEM, FBS under continuous 808 nm laser excitation for 30 min. (a.u. arbitrary unit). Data were shown as mean ± SD, n = 3

**Fig. 2:**
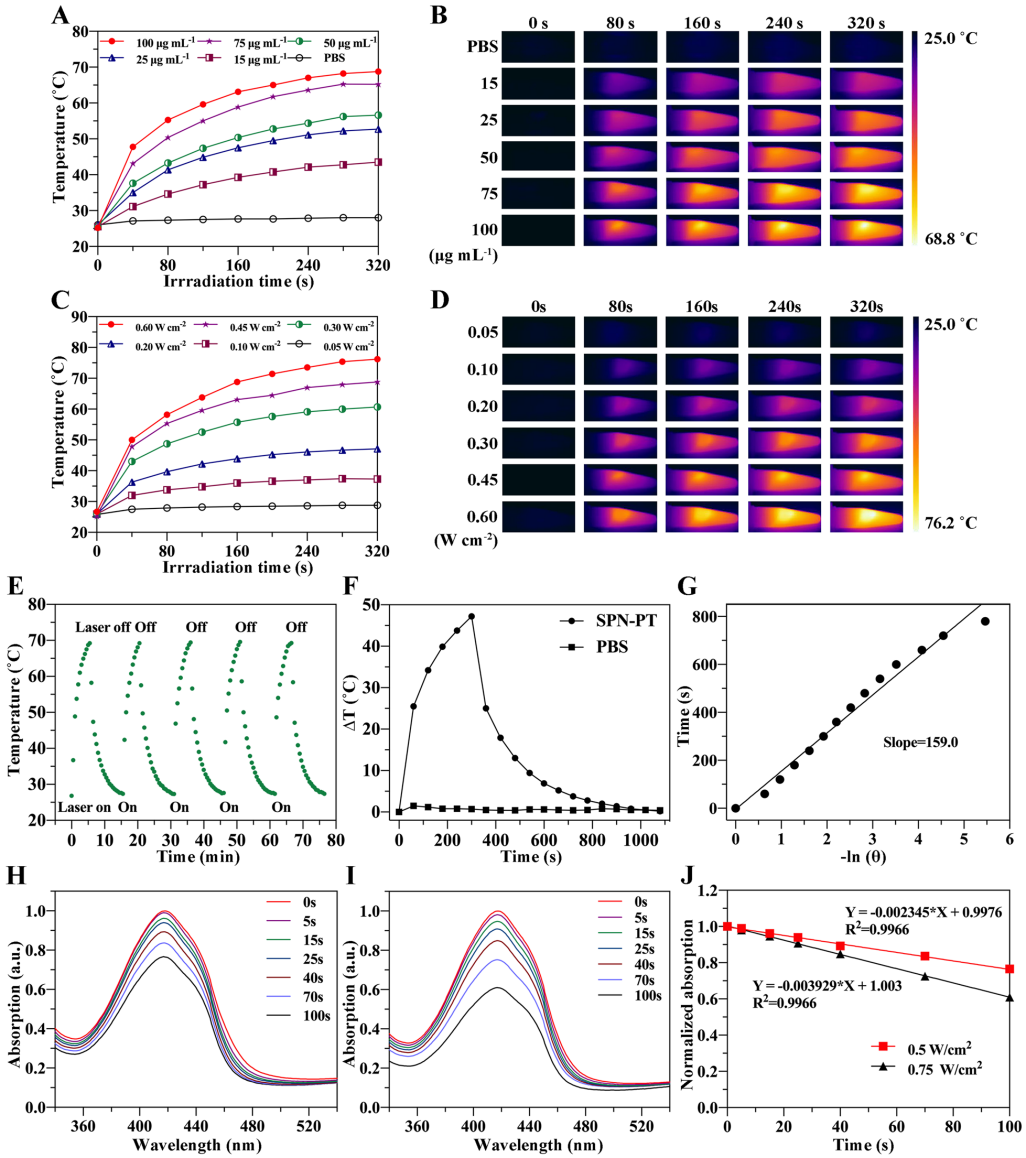
Photothermal and photodynamic performance of SPN-PT. **A** Photothermal conversion behavior of different concentrations of SPN-PT under laser irradiation (635 nm, 0.45 W cm^−2^). **B** IR thermal images of SPN-PT at concentrations corresponding to A irradiated by laser (635 nm, 0.45 W cm^−2^). Temperature elevation curves (**C**) and IR thermal images (**D**) of SPN-PT (100 µg ml^−1^) irradiated by different power densities of laser (635 nm, 0.05 W cm^−2^ - 0.60 W cm^−2^). **E** Temperature change curves of SPN-PT (100 µg mL^−1^) over 5 ON/OFF cycles employing 635 nm laser (0.5 W cm^−2^) followed by passive cooling every 5 min. **F** The photothermal heating and cooling curves of SPN-PT and PBS under the 635 nm laser irradiation (0.6 W cm^−2^). **G** Linear time data versus negative natural logarithm was obtained from the cooling period. Absorption spectra change of DPBF incubated with SPN-PT and irradiated by 635 nm laser at 0.5 W cm^−2^ (**H**) and 0.75 W cm^−2^ (**I**). **J** Absorption intensity changes of DPBF at 419 nm in the presence of SPN-PT (50 µg mL^−1^) with the irradiated time of 635 nm laser (0.5 W cm^−2^, red; 0.75 W cm^−2^, black)

**Fig. 3:**
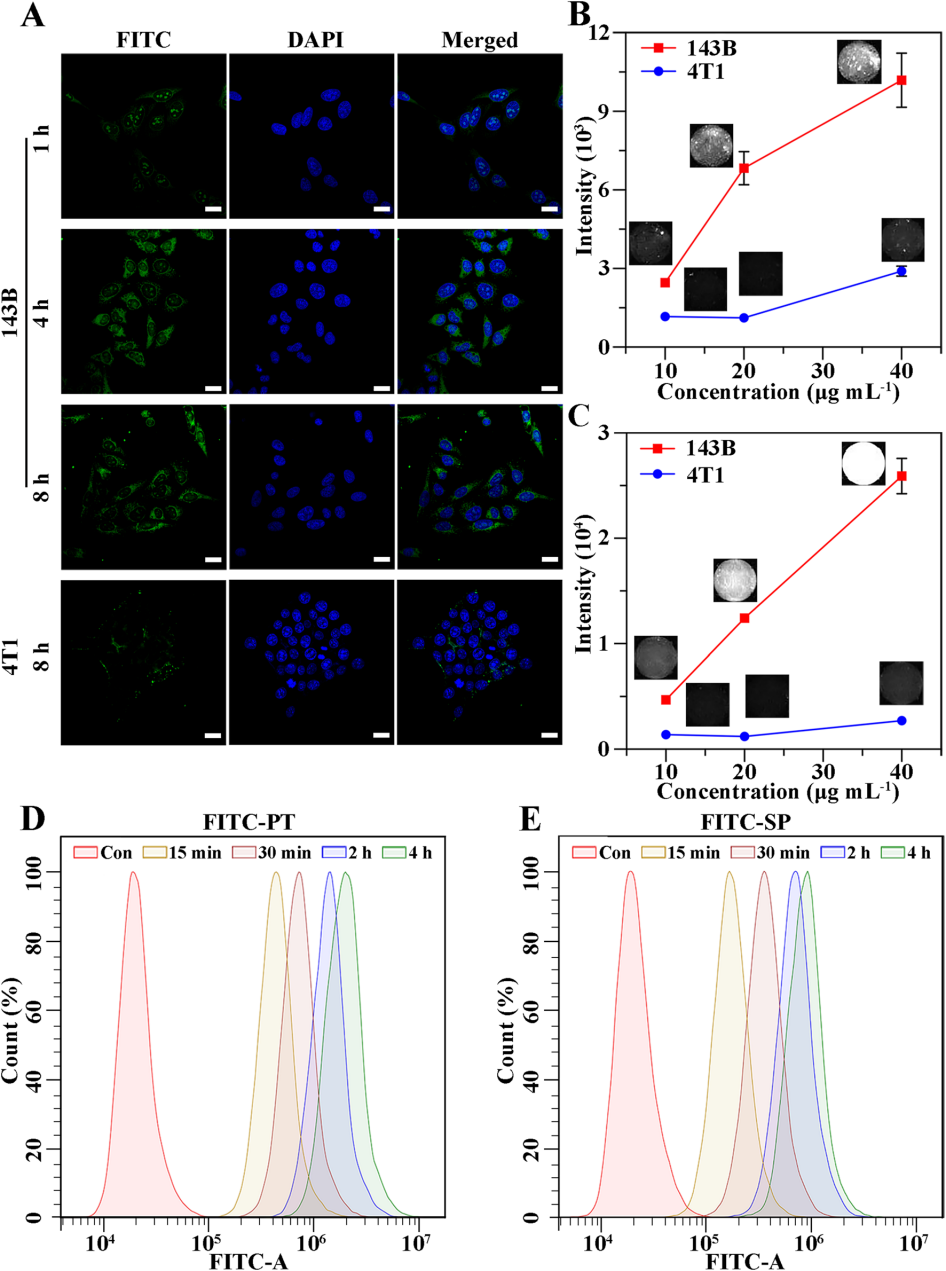
Cellular uptake of FITC-PT and FITC-SP. **A** Representative CLSM images of 143B cells and of 4T1 cells incubated with FITC-PT (20 µg mL^−1^) for different time. Scale bar = 20 μm. Representative images and quantitative statistics of NIR-II fluorescence intensities of 143B or 4T1 cells incubated with different concentrations of SPN-PT (from left to right: 10 µg mL^−1^, 20 µg mL^−1^, 40 µg mL^−1^) for 12 h (**B**) and 24 h (**C**). Flow cytometry analysis of 143B cells incubated with FITC-PT (20 µg mL^−1^) (**D**) and FITC-SP (20 µg mL^−1^) (**E**) for different time (from left to right: Con (control), 15 min, 30 min, 2 h, 4 h). Data were shown as mean ± SD, n = 3

**Fig. 4:**
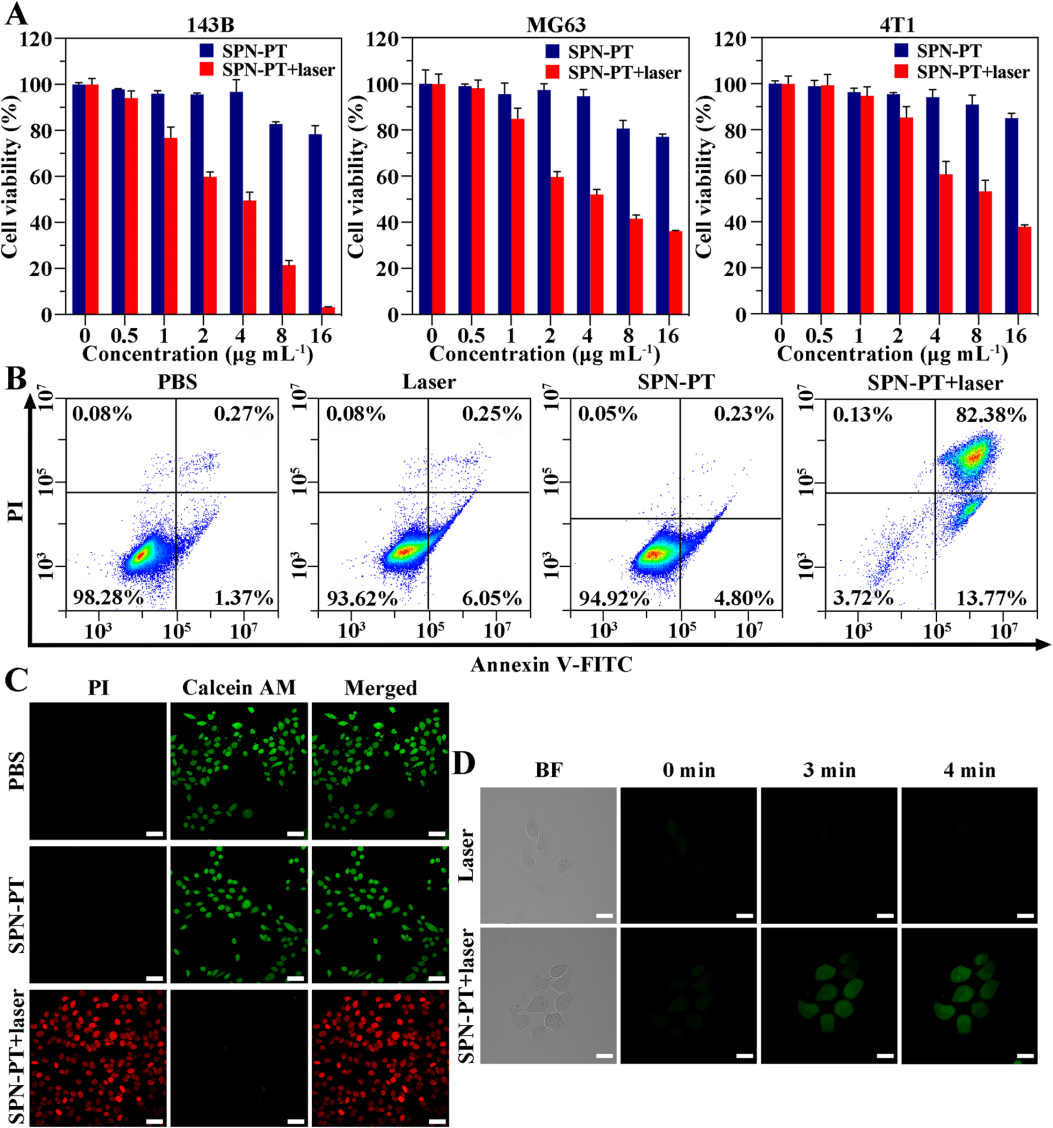
In vitro cytotoxic effects and PDT effects of SPN-PT. **A** Cell viability of 143B, MG63 and 4T1 cells determined by MTT assays after SPN-PT incubation (blue) or SPN-PT+laser treatments (635 nm, 0.75 W cm^−2^, 5 min per well) (red). **B** Flow cytometry of 143B cells co-stained with Annexin V-FITC and PI after different treatments, from left to right: PBS, laser, SPN-PT (16 µg mL^−1^), SPN-PT and laser irradiation (16 µg mL^−1^) (635 nm, 0.5 W cm^−2^, 5 min). **C** Representative CLSM images of live and dead 143B cells received different treatments: PBS (first row), SPN-PT (16 µg mL^−1^) (second row), SPN-PT+laser (16 µg mL^−1^) (635 nm, 0.5 W cm^−2^, 5 min) (third row). Necrotic cell nuclei could be stained by PI in red, while live cells were only stained by Calcein-AM in green. Scale bar = 50 μm. **D** CLSM images of ^1^O_2_ generated by in 143B cells irradiated by 635 nm laser (0.5 W cm^−2^), with (down) or without (up) incubation of SPN-PT for different time. BF: bright field. Scale bar = 20 μm. Data were shown as mean ± SD, n ≥ 3

**Fig. 5:**
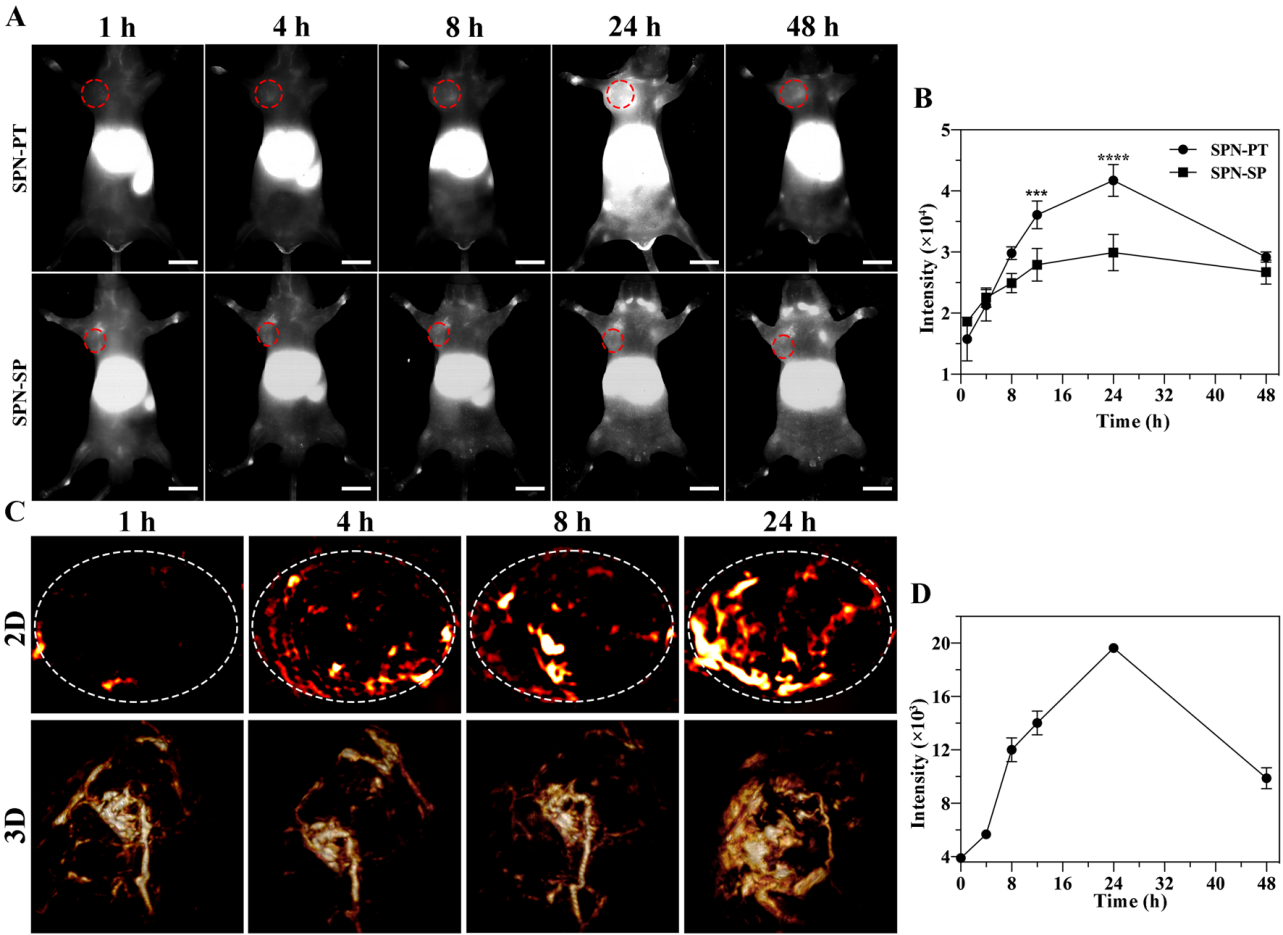
In vivo imaging using SPN-PT. **A** NIR-II fluorescence images of 143B xenograft mice at different time points post injection of SPN-PT (upper, 100 µg mL^−1^, 100 µL, i.v.) or SPN-SP (lower, 100 µg mL^−1^, 100 µL, i.v.). The fluorescence images were acquired under the excitation of 808 nm with a 980 nm filter. Scale bar = 1 cm. **B** Monitoring of NIR-II fluorescence intensities collected within the tumor region (red circle in **A**) of 143B tumor bearing mice. Data were shown as mean ± SD, n = 3. (*p* < 0.05, *; *p* < 0.01, **; *p* < 0.001, ***; *p* < 0.0001, ****). **C** Two-dimensional (upper) and three-dimensional (lower) PA imaging of 143B tumors in living mice injected with SPN-PT (100 µg mL^−1^, 100 µL, i.v.) at 1 h, 4 h, 8 h, 24 h. The PA images were acquired at 695 nm. **D** Average PA intensities within the whole tumor area (white circle in **C**). (i.v. intravenous injection). Data were shown as mean ± SD, n = 3

The properties of SPN-PT were then studied. As shown in Fig. 1A, spherical morphology of SPN-PT was demonstrated by transmission electron microscopy (TEM) imaging, and diameters were determined by dynamic light scattering (DLS) as 124.24 ± 1.34 nm. The stability of SPN-PT was also evaluated by DLS. Dispersed in phosphate buffer solution (PBS), modified Eagle’s medium (MEM), and 10% fetal bovine serum (FBS), SPN-PT did not show any precipitation or obvious change in average diameter even after storage for 30 days, indicating their good physiological stabilities (Fig. 1B). The diameters of SPN-PT decreased in FBS compared to those in PBS or MEM, the possible reason is that the degree of dispersion of SPN-PT may be changed by the biological proteins in FBS. To investigate the photophysical behavior, the absorbance and fluorescence spectra of SPN-PT in PBS solution were studied. SPN-PT had an intense UV absorbance around 703 nm while fluorescence emission peaked at 961 nm and extended into NIR-II region (Fig. 1C). A linear correlation between the concentration of SPN-PT and its absorption (Fig. 1D) and emission (Fig. 1E; Additional file 1: Figure S6A, B) was observed. Then the PA imaging capability of SPN-PT was investigated. The PA spectra were performed from 680 to 820 nm using a PA imaging system and SPN-PT exhibited the highest PA signal at the wavelength of 695 nm (Fig. 1F). The intensity of PA (Fig. 1G) also demonstrated a positive correlation with the concentration of SPN-PT aqueous solutions. Photobleaching study demonstrated a better photostability of SPN-PT compared with that of chlorin e6 (Ce6) (Fig. 1H). The absorption of SPN-PT merely changed within 30 minutes’ irradiation, while that of Ce6 decreased by 36.5%. Moreover, the fluorescence emission intensities of SPN-PT in the three solvents also displayed negligible decline throughout the continuous laser irradiation for 30 min (Fig. 1I), revealing a favorable fluorescence stability of SPN-PT. These results demonstrate favorable stabilities of SPN-PT, which may benefit from the stable structures of its each component, namely DSPE-PEG_2000_-COOH and PCPDTBT. In addition, PCPDTBT has large molecular weight and strong hydrophobicity. As a result, it will not easily penetrate from the hydrophobic core of the nanoparticles like other small molecules. The structural stability of nanoparticles containing PCPCTBT has also been revealed in other researches [37, 38]. These results above revealed that SPN-PT was a good candidate for NIR-II fluorescence/PA imaging.

### PTT and PDT effects of SPN-PT

Encouraged by the NIR absorption of SPN-PT, the photothermal effects of SPN-PT were estimated under a 635 nm laser. As illustrated in Fig. 2 A, B, the PBS solution of SPN-PT exhibited remarkable rise of temperature in a concentration-dependent manner under a 635 nm laser of 0.45 W cm^−2^. Under the irradiation of the laser, the maximum temperature of the solution rapidly rose to 68.8 ºC (Δ*T* = 43.6 ºC) within 5 min when the concentration of SPN-PT reached 100 µg ml^−1^ (Fig. 2A). By contrast, scarcely had the temperature increased in PBS under the same irradiation. Furthermore, the plateau temperature of 100 µg mL^−1^ of SPN-PT revealed a positive correlation with the power density of laser (Fig. 2C, D). Subjected to repeated heating-cooling cycles for 75 min, SPN-PT steadily rose to almost the same plateau temperature (69.34 ± 0.17 ºC) after every 5 minutes’ irradiation (635 nm, 0.5 W cm^−2^) (Fig. 2E), illustrating an excellent stability of the material under 635 nm laser irradiation (0.5 W cm^−2^). Exposed to a laser at 0.6 W cm^−2^, the temperature of SPN-PT can even reach 76.2 ºC (Δ*T* = 49.5 ºC), indicating a favorable photothermal conversion character of SPN-PT. The photothermal conversion efficiency (*η*) of SPN-PT was calculated to be 41.8% based on the heating-cooling cycle according to the reported methods (Fig. 2F, G) [39]. The PDT efficacy of SPN-PT (50 µg mL^−1^) under different power densities of 635 nm laser irradiation were also measured with a utilization of 1,3-diphenylisobenzofuran (DPBF) probe, of which the absorbance correspondingly decreases in the presence of singlet oxygen (^1^O_2_) produced by SPN-PT [40]. As depicted in Fig. 2H, I, the absorbance of DPBF at 419 nm dropped from 1.0 to 0.765 and 0.609 after 100 seconds’ irradiation of laser respectively at 0.5 W cm^−2^ and 0.75 W cm^−2^ power densities. Moreover, the absorbance of DPBF decreased with the increase of irradiation time, as summarized in Fig. 2J. These data indicated the good photothermal and photodynamic conversion efficiency of SPN-PT.

### Cell uptake of SPN-PT and SPN-SP

For further biological applications, the uptake specificity and efficiency into osteosarcoma cell lines of SPN-PT were firstly explored before estimation of its capability in tumor imaging and therapies. As the emission wavelength of SPN-PT and SPN-SP was too long for confocal imaging, fluorescein isothiocyanate (FITC) were chosen as the fluorescence producer at the wavelength of 520 -530 nm to dope into the nanoparticles, named as FITC-PT or FITC-SP. Firstly, FITC-PT was incubated with 143B, MG63 or 4T1 cells for different durations before confocal laser scanning microscopy (CLSM) imaging was employed. As described in Fig. 3A and Additional file 1: Figure S7, the fluorescence signal increased with the incubation time of FITC-PT with OS cells, and intense signal was observed after incubation for 4 h (Fig. 3A, second row; Additional file 1: Figure S7, second row). On the contrary, 4T1 cells did not show much fluorescence signal after 8 h’s incubation with FITC-PT, indicating the excellent selectivity of nanoparticles equipped with peptide targeting to osteosarcoma cells rather than other tumor cells. In addition, the uptake of SPN-PT was also verified via NIR-II fluorescence imaging. 143B cells or 4T1 cells were co-cultured with SPN-PT of multi-concentrations for 1 h (Additional file 1: Figure S8A), 4 h (Additional file 1: Figure SB), 12 h (Fig. 3B) or 24 h (Fig. 3C). Seeing from the representative NIR-II fluorescence images and corresponding quantitative intensities, SPN-PT successfully lighted 143B cells up in concentration-dependent and time-dependent manners, with more efficient uptake and intense signals compared to 4T1 cells. To specify uptake efficiency of the nanoparticles into 143B cells, flowcytometry assessments were applied after different incubation of time. FITC-PT and FITC-SP were respectively incubated with 143B cells for 15 min, 30 min, 2 h and 4 h. FITC-PT demonstrated a rapid uptake into cells within 4 h whereas the uptake of FITC-SP was lower under the same condition (Fig. 3D, E). These results confirmed a faster uptake into the targeted cells for FITC-PT. In a word, the good selectivity and efficiency of cellular uptake into 143B cells can be attributed to the OS-targeted peptide PT.

### In vitro therapeutic effects of SPN-PT

Based on the superior cellular uptake, the therapeutic effects of SPN-PT in different cell lines were further explored. 3-(4,5-dimethylthiazol-2-yl)-2,5-diphenyltetrazolium bromide (MTT) assays were performed to evaluate in vitro biocompatibility and phototoxicity of SPN-PT. Respectively cultured with 143B, MG63 and 4T1 cells, SPN-PT showed negligible cytotoxicity without laser irradiation, demonstrating the good biocompatibility (Fig. 4A). Being irradiated by 635 nm laser, 143B, MG63 and 4T1 cells all emerged with distinct decrease of viabilities. Meanwhile, the cell viability decreased with the increase of SPN-PT concentrations, and the viabilities of 143B cells incubated with the highest concentration of SPN-PT (16 µg mL^−1^) even dropped to 3.14%, reflecting an efficient phototoxicity of SPN-PT for 143B cells. To further verify the apoptosis of 143B cells, flow cytometry assays with Annexin V-FITC/ propidium iodide (PI) staining were performed after different treatments. Acquired quantitative results revealed a majority of late apoptotic cells (82.38%) and part of early apoptotic ones (13.77%) in cells treated by SPN-PT and laser irradiation, while live cells composed the most of other groups (93.62% - 98.28%) (Fig. 4B). In addition, CLSM imaging was employed to further investigate the therapeutic effects of SPN-PT in vitro. The treated 143B cells were bi-labelled by calcein-acetoxymethyl (Calcein-AM), indicating live cells in green, and PI, emitting red fluorescence in dead cells. As shown in Fig. 4C, the 143B cells that received no treatments or the cells treated with SPN-PT without laser irradiation exhibited simply green fluorescence, indicating no obvious cytomembrane rupture was found. Whereas cells treated with SPN-PT under 5 min laser irradiation showed almost all red fluorescence in the field, describing the death of the cells. These results affirmed the anti-tumor effects in vitro driven by PTT/PDT therapy of SPN-PT in multiple aspects. The PDT effects of SPN-PT in vitro were also checked with the application of 2’,7’-dichlorodihydrofluorescin diacetate (DCFH-DA) kit for detection of ^1^O_2_ generated in cells. 143B cells were incubated with SPN-PT (16 µg mL^−1^) for 8 h before 635 nm laser irradiation (0.5 W cm^−2^) were applied. The CLSM imaging was employed immediately after every minute of irradiation. Obvious ^1^O_2_ could be observed in 143B cells from 3 min’s irradiation (Fig. 4D).

#### In Vivo NIR-II fluorescence/PA Imaging using SPN-PT

Based on the good biocompatibility and targeting property of SPN-PT, in vivo NIR-II fluorescence and PA imaging were carried out on 143B xenograft bearing mice to evaluate the targeted imaging ability of SPN-PT. To estimate the merits of NIR-II fluorescence imaging in angiography, NIR-II fluorescence image of blood vessels from mice with143B tumor were captured under 808 nm excitation with 980 nm filter immediately after intravenous injection of SPN-PT (100 µg mL^−1^, 100 µL). The vascular of hind limb and fore limb could be clearly discerned from the background and the signal-to-background ratios (SBR) were respectively calculated as 7.752 and 4.274 in fore limb and hind limb (Additional file 1: Figure S9). These results demonstrated that SPN-PT is equipped with favorable optical spatial resolution and contrast, providing a high quality of in vivo NIR-II fluorescence imaging. As a result, continuous NIR-II fluorescence (Fig. 5A) and PA imaging (Fig. 5C) were performed in 143B xenograft bearing mice, and their intensities in the region of tumor were dynamically monitored at different time points (1, 4, 8, 24 and 48 h) after intravenous injection of SPN-PT (100 µg mL^−1^, 100 µL) or SPN-SP (100 µg mL^−1^, 100 µL). As depicted in Fig. 5A, B, the NIR-II fluorescent intensity in the tumor region for SPN-PT-treated mice increased faster than that of SPN-SP-treated mice, and significantly higher intensity was observed at t = 12 (*p* < 0.001, ***) and 24 h (*p* < 0.0001, ****) post-injection. These results revealed the better accumulation ability of SPN-PT than SPN-SP. Similarly, PA intensities increased at the region of xenograft from 4 to 8 h after injection and reached the peak within 24 h. The difference was that the NIR-II fluorescence intensity arose earlier at about 4 h (Fig. 5A, B), illustrating the sensitivity of NIR-II fluorescence imaging. Whereas PA imaging exhibited more details of the tumor, namely, the vascular as well as the refined structures of tumor could be lighted up (Fig. 5C; Additional file 2: Movie S1, Additional file 3: Movie S2, Additional file 4: Movie S3 and Additional file 5: Movie S4). These results demonstrated the advantages of the dual-modal nanoprobe SPN-PT, involving the sensitive property which enables an earlier diagnosis for tumors, and the high temporal and spatial resolution which guarantees a detailed diagnosis as well as possibilities for providing intraoperative guiding and helping accurate tumor resections.

The metabolism and biodistribution were subsequently defined. The concentrations of SPN-PT in the blood circulation were determined by NIR-II fluorescent intensities of serum samples collected from mice at different time points after intravenous injection of SPN-PT (5 min, 15 min, 30 min, 45 min, 1 h, 2 h, 4 h, 6.5 h, 8 h, 12 h, 24 h) in accordance to a standard curve (Additional file 1: Figure S10A) acquired using the same heparinized capillary under the same imaging conditions. The half-life (t_1/2_) of SPN-PT in serum was calculated to be ~ 0.3198 h (Additional file 1: Figure S10B), implying the serum level of SPN-PT could reduce by half within 19 min. Moreover, the biodistribution of SPN-PT was explored via NIR-II fluorescence imaging of major organs and tumors excised from mice 48 h post injection. As showed in Additional file 1: Figure S11A, B, brighter fluorescence was mainly observed in the tumor, liver and spleen.

### In vivo therapies of SPN-PT

**Fig. 6:**
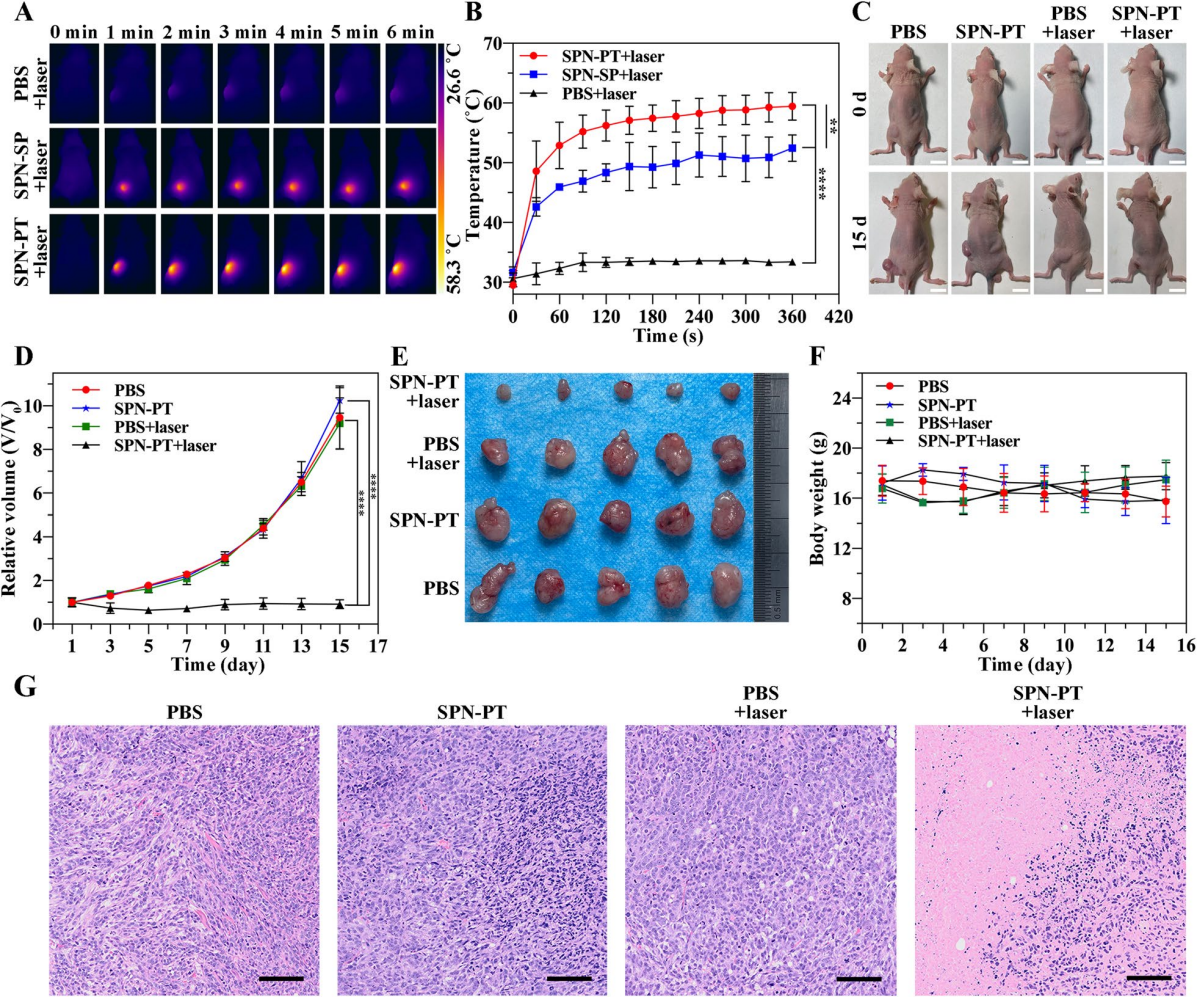
SPN-PT-based therapeutic effects. **A** IR thermal images of 143B xenograft mice treated with PBS + 635 nm laser (0.75 W cm^−2^) (upper row), SPN-SP (100 µg mL^−1^, 100 µL) + 635 nm laser (0.75 W cm^−2^) (second row), and SPN-PT (100 µg mL^−1^, 100 µL) + 635 nm laser (0.75 W cm^−2^) (lower row) for 6 min. **B** Corresponding temperature changes in the region of tumors from (A). **C** Representative morphology images of 143B tumor bearing mice received different treatments on 0 d (upper row) and 15 d (lower row). Scale bar = 1 cm. Monitoring of tumor volumes (**D**) and body weight of mice **F** after application of different treatments within the 15 days’ therapeutic procedure. **E** Representative photograph of tumors excised from mice on 15 d post treatments. **G** H&E results of tumors obtained from four groups on 24 h post treatments. Scale bar = 200 μm. Data were shown as mean ± SD, n ≥ 3. (*p* < 0.05, *; *p* < 0.01, **; *p* < 0.001, ***; *p* < 0.0001, ****)

To study the therapeutic efficiency of SPN-PT in vivo, subcutaneous 143B xenograft mouse model was established and applied with PTT/PDT therapies driven by PBS/SPN-SP/SPN-PT. In accordance with the intensity monitoring results of NIR-II fluorescence and PA imaging, PTT therapy was employed 24 h after intravenous injection of PBS/SPN-SP/SPN-PT using a 635 nm laser at 0.75 W cm^−2^. The mice with 143B tumors received systemic administration of PBS (100 µL), SPN-SP (100 µg mL^−1^, 100 µL), SPN-PT (100 µg mL^−1^, 100 µL), and were followed by 635 nm laser irradiation (0.75 W cm^−2^, 6 min) at 24 h after the injection. Seeing from the photothermal images in Fig. 6A, B, the PTT driven by SPN-PT+laser revealed a dramatically faster increase of temperature compared with that of PBS+laser and that of SPN-SP+laser, which as a result, arrived at a higher plateau temperature (58.3 °C; *p* < 0.0001) at the end of PTT therapy. By contrast, accumulating in osteosarcoma tumors in a passive way rather than targeted aggregation, SPN-SP exhibited weaker PTT effects at the tumor region but still reached a significant higher plateau temperature (50.9 °C; *p* < 0.0001) compared with that of PBS+laser (34.1 °C). These results verified the increase of temperature in mice treated with SPN-PT+laser and SPN-SP+laser, impelling subsequent exploration of the therapeutic effects of SPN-PT in inhibiting tumor growth. The PDT effects in vivo were verified using Singlet Oxygen Sensor Green (SOSG) probe [41]. As shown in Additional file 1: Figure S12, tumor tissues from mice treated with SPN-PT (100 µg mL^−1^, 100 µL) and laser irradiation (635 nm, 0.75 W cm^−2^) for 10 min (third row) showed significant generation of singlet oxygen, while those treated with SPN-SP (100 µg mL^−1^, 100 µL) and laser irradiation (635 nm, 0.75 W cm^−2^) (the last row) showed weaker fluorescence signals. These results verified the PDT effects of SPN-PT in vivo and further proved higher therapy effects of SPN-PT compared to that of SPN-SP, which may benefit from the more efficient accumulation of nanoparticles with targeting ability.

**Fig. 7:**
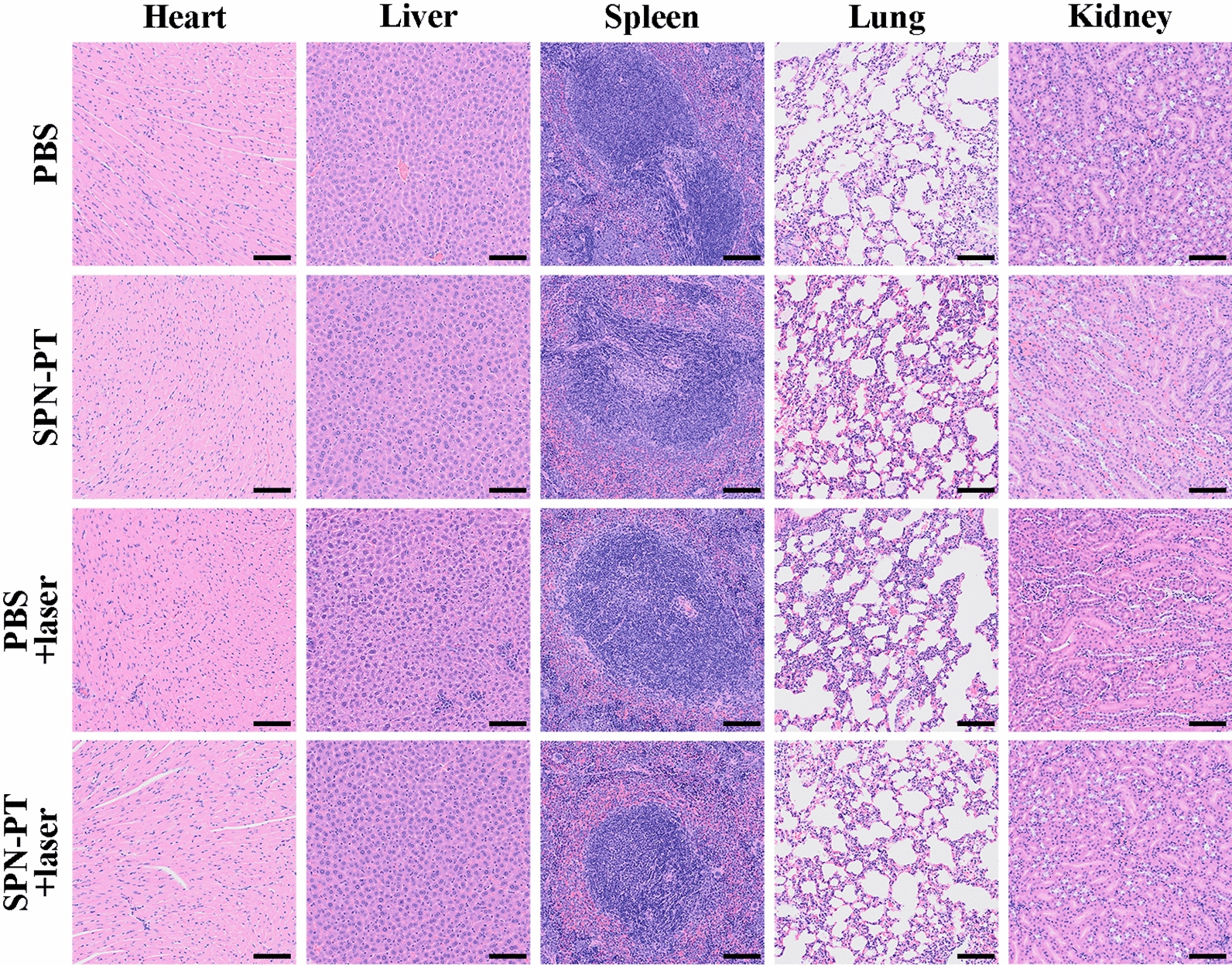
Biocompatibility evaluation of SPN-PT-based therapies. H&E staining of major organs (heart, liver, spleen, lung, kidney) excised from mice at the endpoint of the experimental therapies. Scale bar = 200 μm

Thus, a number of nude mice were inoculated with 143B cells subcutaneously, and those with a similar volume of the tumor were randomly divided into 4 groups (n = 5 per group) on 14 days after the inoculation. Two groups received injection of PBS (100 µL) and with or without subsequent treatment of laser illumination. While the other two groups were administrated with SPN-PT (100 µg mL^−1^, 100 µL) and with or without laser illumination of the same wavelength and power density. Those mice who received laser treatment were exposed to 635 nm laser (0.75 W cm^−2^) for 10 min. The mice were monitored in the following 15 days, with their tumor volumes and body weights recorded carefully every other day. Seeing from the overall picture taken before therapy (0 d) and at the end of the therapy (15 d) (Fig. 6C), there was a remarkable difference in tumor sizes of mice in SPN-PT+laser treated group, compared with those in other three groups on 15 d, while the sizes before the employment of treatments were less differentiating. As shown in Fig. 6D, the tumors grew fast in other three groups except for those treated with SPN-PT+ laser. For those SPN-PT injected and laser irradiated mice, the tumor volume even had a slight decrease in the first week and had negligible increase afterwards. Figure 6E visibly displayed all the tumors at the therapeutic end, also described the excellent inhibition effects carried out by the SPN-PT-based PTT therapy. All the mice had a normal body weight throughout the therapy and inacceptable loss of body weight had not been found in any mice (Fig. 6 F), showing a favorable biocompatibility of SPN-PT. Biocompatibility were also confirmed through hematoxylin and eosin (H&E) staining of major organs (heart, liver, spleen, lung and kidney) resected from mice of different groups at the end of therapeutic process (15 d). Figure 7 revealed no obvious necrosis, no inflammatory edema, no structural damage in major organs from histological level, additionally validated the biocompatibility of the PTT therapy driven by SPN-PT. Additionally, serum samples collected from the mice at the end of different treatments further confirmed that there was no damage on liver and renal functions (Additional file 1: Figure S13). These results affirmed the feasibility of SPN-PT-based therapy in vivo.

To further investigate the anti-tumor effects at a histological level, 143B xenografts were collected and fixed for H&E staining on 24 h after PTT therapy was employed. As illustrated in Fig. 6G, significant disorganization of cell arrangement and regional necrosis were found in tumor tissues treated by SPN-PT administration and laser irradiation, while no obvious histological changes were found in tumors from other groups, further demonstrating the superior therapeutic efficacy of SPN-PT in OS tumors.

## Conclusions

In summary, we developed a peptide-based osteosarcoma-targeted SPNs (SPN-PT) for dual-modal imaging (NIR-II fluorescence/NIR-I PA) guided phototherapies (PTT/PDT). The peptide was incorporated into amphiphilic copolymer (DSPE-PEG_2000_-COOH) to provide water solubility for biocompatible applications. The NIR-absorbing semiconducting polymer (PCPDTBT) was used as the source of NIR-II fluorescence and PA signals, and served as the PS for PTT and PDT. SPN-PT was formulated through nanoprecipitation method with a size of about 124 nm. The feasibility and efficiency of SPN-PT as PS has been investigated in detail, in terms of physiochemical characters, PTT and PDT performance, and preliminary cell-based studies and in vivo therapies. In particular, the cellular uptake of the peptide driven nanoparticles into targeted OS cells is far more rapid than control, which can be finished within 4 h. Collectively, due to the peptide-based targeting ability, SPN-PT demonstrated selective and efficient OS cellular uptake and boosted strong NIR-II fluorescence and PA signals offering high-sensitivity and high-resolution images for OS tumors. Simultaneously, the enhanced cell accumulation contributed to effective intratumor distributed phototherapies. Our study predicts a promising peptide-based theranostic platform for OS early diagnosis and precise therapies. Besides semiconducting polymers, other kinds of fluorophores can also be encapsulated into nanoplatforms for different imaging modalities such as NIR-II PA imaging. In addition, anticancer drugs may be loaded into the nanoparticles to endow the SPNs with chemo/photo combination therapy to further promote the therapeutic efficacy.

## Materials and methods

### Reagents

1,2-distearoyl-sn-glycero-3-phosphoethanolamine-N-[carboxy(polyethylene glycol)] (DSPE-PEG_2000_-COOH) was purchased from Ponsure Biological (PS2-CMDE-2 K, Mw = 2000, Purity: 95%). Poly[2,6-(4,4-bis-(2-ethylhexyl)-4 H-cyclopenta [2,1-b;3,4-b′]dithiophene)-alt-4,7(2,1,3-benzothiadiazole)] (PCPDTBT) was purchased from Sigma-Aldrich (St. Louis, MO). Peptide PT (PPSHTPT) and control peptide SP (STPHPTP) were purchased from GL biochem Ltd. Tetrahydrofuran (THF) were freshly distilled from sodium/benzophenone, N,N-dimethylformamide (DMF) and dichloromethane (CH_2_Cl_2_) were distilled from CaH_2_. All other standard reagents were purchased from commercial suppliers (such as Sigma-Aldrich) and used without further purification. Annexin V-FITC/PI cell apoptosis kit and MTT were obtained from Key Gene Tech Co. (Shanghai, China).

### Synthesis of PEG-PT and PEG-SP

Synthesis of PEG-PT and PEG-SP was performed following procedures as below: DSPE-PEG_2000_-COOH (30 mg, 15 µmol), 1-[bis(dimethylamino)methylene]-1 H-1,2,3-triazolo[4,5-b]pyridinium 3-oxid hexafluorophosphate (HATU) (6.84 mg, 18 µmol), and hydroxybenzotriazole (HOBT) (2.43 mg, 18 µmol) were dissolved in 3 mL dry N,N-Dimethylformamide (DMF) under an argon atmosphere. Then the mixture was stirred at room temperature for 30 min. Subsequently, peptides PT or SP (10 mg, 13.56 µmol) were dissolved in dry DMF together with N,N-Diisopropylethylamine (DIPEA) (1.8 mg, 14 µmol) and were added to the fore reaction solution and stirred overnight at room temperature to obtain PEG-PT and PEG-SP.

### Fabrication of nanoparticles

Conventional nanoprecipitation method was applied to fabricate SPN-PT and SPN-SP nanoparticles [36]. PCPDTBT (0.5 mg), as well as PEG-PT or PEG-SP (5 mg) were dissolved in 0.5 mL THF, and injected into 5 mL phosphate buffer saline (PBS) as fast as possible, under continuous vigorous sonication for 3 min. The acquired nanoparticle solutions were blown by gentle nitrogen flow to eliminate the THF, then SPN-PT and SPN-SP were obtained.

To construct nanoparticles doped with FITC, 0.1 mg of PCPDTBT, 5 mg of PEG-PT or PEG-SP and 40 µg of FITC were dissolved by 0.3 mL THF and injected into 5 mL PBS, being sonicated for 3 min. The fabricated nanoparticles were termed as FITC-PT or FITC-SP.

### Photostability

Photobleaching study was performed to compare the photostability of SPN-PT with Ce6. Ce6 and SPN-PT (100 µg mL^−1^) were respectively irradiated by 635 nm laser (0.75 W cm^−2^). The absorption was evaluated at different time post irradiation (0 min, 5 min, 10 min, 15 min, 20 min, 25 min, 30 min).

### Photothermal effect and photothermal conversion efficiency

Temperature changes of SPN-PT in PBS (200 µL) were tested in two forms, one of which was that various concentrations of SPN-PT (PBS or 15, 25, 50, 75 and 100 µg mL^−1^) were irradiated by 635 nm laser at 0.45 W cm^−2^. Otherwise, the SPN-PT solution with a concentration of 100 µg mL^−1^ was irradiated with 635 nm laser at gradient power densities (0.05, 0.1, 0.2, 0.3, 0.45 and 0.6 W cm^−2^). The temperature was monitored by an IR thermal camera every 40 s within the 5 minutes’ irradiation of laser.

Subsequently, the photothermal conversion efficiency (*η*) of SPN-PT was explored using a thermal imaging camera (Fotric 225, Fotric Precision Instruments, USA, ± 2 °C). The aqueous solution of SPN-PT (100 µg mL^−1^, 200 µL) was irradiated with 635 nm laser at 0.6 W cm^−2^ for 5 min before the laser was removed, and then a temperature increase and drop curve was drawn based on the data acquired throughout this procedure. Thus, the photothermal conversion efficiency (*η*) was calculated according to Eqs. (1) and (2) as below:

1 $$\eta =\frac{hs\left({T}_{max}-{T}_{surr}\right)-{Q}_{dis}}{I\left(1-{10}^{-{A}_{\lambda }}\right)}$$

2 $${\tau }_{s}=\frac{{m}_{D}{C}_{D}}{hS}$$

The parameters *S*, *h*, *T*_*max*_, *T*_*surr*_, *Q*_*dis*_, *I* and *A*_*635*_ are the container’s surface area, heat-transfer coefficient, laser triggered maximum temperature, surrounding temperature, heat dissipation caused by the light absorbing of quartz cuvette, intensity of laser and absorbance of SPN-PT (100 µg mL^−1^) at 635 nm, respectively. Parameter *τ*_*s*_ is the time constant of the sample system. The parameters *m*_*D*_ and *C*_*D*_ are respectively the mass and heat capacity of the solvent.

To further evaluate the photothermal stability of SPN-PT, 100 µL of SPN-PT aqueous solution (100 µg mL^−1^) were subjected to repeated heating-cooling cycles for 75 min (5 cycles). In each heating-cooling, SPN-PT was irradiated by 635 nm laser (0.5 W cm^−2^) for 5 min and was followed by passive cooling for 10 min. All the temperature changes of SPN-PT solutions were performed with an IR thermal camera, and these data were recorded every 30 s.

### Cell line and animal model

4T1 cells (murine mammary carcinoma cell line) were purchased from the Shanghai Laboratory Animal Center, Chinese Academy of Science (SLACCAS). 143B, MG63 (human osteosarcoma cell lines) cells were purchased from American Type Culture Collection (ATCC). Supplemented with 10% fetal bovine serum (FBS) (Gibco, Grand Island, NY, USA), 143B, MG63 cells were cultured with MEM (Gibco, Grand Island, NY, USA) and 4T1 cells were cultured in Dulbecco’s Modified Eagle’s Medium (DMEM, Gibco, U.S.), in addition with 1% (v/v) penicillin-streptomycin at 37 °C in a humidified incubator containing 5% CO_2_. The 143B xenograft tumor models were established by subcutaneous injection of 143B cells (∼5 × 10^6^ in 150 µL of PBS) into Balb/c nude mice (male, 3–5 week, 10–15 g, Yangzhou University Comparative Medicine Centre) under anesthesia using isoflurane. All animal procedures in this study were approved by the Ethics committee of Zhongnan Hospital of Wuhan University, Wuhan, China (ZN2021062).

### Measurement of singlet oxygen generation and DCFH-DA assay

DPBF probe was employed for extra cellular ^1^O_2_ generated by SPN-PT. SPN-PT aqueous solution (100 µg mL^−1^, 1 mL) was mixed with 400 µL of ethanol solution containing DPBF (10 mM). Then the solution was kept in dark and thoroughly stirred. The absorbance of DPBF in the solution at 419 nm was measured by UV-vis spectra within 100 s’ irradiation (635 nm, 0.5 W cm^−2^ or 0.75 W cm^−2^) (0 s, 5 s, 15 s, 25 s, 40 s, 70 s, 100 s).

143 B cells (1 × 10^4^ cells/well) were seeded onto a 15 mm confocal dish and incubated with 16 µg mL^−1^ SPN-PT for 8 h and 10 µM DCFH-DA (*Ex*/*Em* = 504/529 nm) for 30 min. The cells were then exposed to laser irradiation (0.5 W cm^−2^, 635 nm) for 4 min. Throughout the irradiation, tumor cell samples were examined by the laser scanning confocal microscope every minute, using a high-pressure mercury lamp as excitation source. Fluorescence was collected using an excitation wavelength of 488 nm and recording the emission at 500–550 nm (green).

### MTT assay

In vitro cytotoxicity studies of SPN-PT on 143B cells, MG63 cells and 4T1 cells were performed by using MTT cytotoxicity assay. Cells were seeded in 96-well plates at densities between 5000 and 10,000 cells per well. Then the cells were incubated with 100 µL of fresh cell media containing SPN-PT for 24 h. The final concentrations of SPN-PT in the culture medium were fixed at 0, 0.5, 1, 2, 4, 8, 16 μg mL^−1^. Then MTT (0.5 mg mL^−1^) was added into each well (20 µL) and incubated at 37 °C for 4 h. After that, using dimethyl sulfoxide (DMSO) to resolubilize the formazan converted by MTT. The absorbance was measured at 490 nm using Bio-Tek Synergy HTX. The following formula was used to calculate the viability of cell growth: Cell viability (%) = (mean of absorbance value of treatment group/mean of absorbance value of control) × 100.

To investigate the phototoxicity of SPN-PT on different cells, similar amounts of 143B cells were seeded and then incubated with varied concentrations of SPN-PT for 24 h, subsequently treated with laser irradiation (635 nm, 0.75 W cm^−2^, 5 min per well). And MTT analyses were employed 24 h after the irradiation.

### Cellular uptake evaluated by CLSM, flow cytometry and NIR-II fluorescence imaging

FITC doped nanoparticles FITC-PT were used in CLSM imaging. 5 × 10^4^ cells per well of 143B or 4T1 cells were seeded onto confocal dishes for CLSM or and cultured overnight in MEM or DMEM supplemented with FBS (10%) and penicillin-streptomycin (1%) at 37 °C in a humid air atmosphere containing 5% CO_2_. Then culture media was replaced with 1.5 mL of MEM or DMEM containing FITC-PT (20 µg mL^−1^) and respectively cultured for 1 h, 4 h, 8 h before CLSM imaging. Moreover, 4’,6-diamidino-2-phenylindole (DAPI) was further incubated with cells for 10 min for nuclear staining. After that, the cells were triply washed with PBS and applied to CLSM.

In terms of flow cytometry analyses, 5 × 10^4^ cells per well of 143B cells were seeded onto 6-well plates, and cultured overnight before incubation with FITC-PT or FITC-SP (15 min, 30 min, 2 h, 4 h) (20 µg mL^−1^). After the incubation and washing, the cell samples were collected with 0.05% of trypsin, the fluorescence intensity of the cells was analyzed using a flow cytometer.

To compare the cellular uptake of SPN-PT in 143B and 4T1 cells via NIR-II fluorescence imaging, 5 × 10^4^ cells per well of 143B or 4T1 cells were seeded to 6-well plates for 12 h, thus incubated with varied concentrations of SPN-PT (final concentration were adjusted to 10 µg mL^−1^, 20 µg mL^−1^, 40 µg mL^−1^) for different durations (12 h, 24 h). At the experimental end, cells were thoroughly washed with PBS and imaged using a NIR-II fluorescence imaging system. The cell samples were excited by an 808 nm laser and fluorescence signals were collected through a 980 nm filter.

### Apoptosis or necrosis of 143B cells through Annexin V-FITC/PI staining and Calcein-AM/PI staining

143B cells were seeded in 6-well plates at a density of 5 × 10^4^ cell per well. Cells were cultured overnight and incubated with SPN-PT (16 µg mL^−1^) for 12 h, followed by laser irradiation to induce cell death (635 nm, 0.5 W cm^−2^, 5 min per well). 12 h after treatments, the cells were harvested and re-suspended in binding buffer, stained with Annexin V-FITC and PI according to the manufacturer’s instructions, then administered to flow cytometer for fluorescence intensity collection and analyses.

Besides, we also used Calcein-AM and PI dual staining to evaluate the cellular apoptosis and necrosis. Similarly, 143B were treated the same as described above and followed by co-staining of Calcein-AM/PI on 24 h post treatments. Normal cell nuclei could only be stained by Calcein-AM (green), while necrotic cell nuclei would be stained by PI (red) as a result of cytomembrane rupture.

### Photoacoustic Imaging (PAI)

The photoacoustic signals were recorded using a Nexus 128 photoacoustic instrument (Endra Inc., Boston, MA) with a series of laser wavelengths in the range of 680-820 nm. The PA data are reconstructed in volumes of 768 × 768 × 768 with 0.1 × 0.1 × 0.1 mm voxels. The system is equipped with a tunable nanosecond pulsed laser (wavelength-dependent laser power density, about 2-3 mJ pulse^−1^ on the animal surface) and 128 unfocused ultrasound transducers (with 5 MHz center frequency and 3 mm diameter) arranged in a hemispherical bowl filled with water (temperature is set to 38 °C). The imaging data were analyzed using Osirix software (Pixmeo SARL, Bernex, Switzerland). The aqueous solution of SPN-PT was filled into polyethylene centrifuge tube for PA imaging.

For in vivo imaging, the 143B tumor-bearing mouse anesthetized with 2% isoflurane in oxygen was placed in the imaging tray at an appropriate position within the focal field of view (FOV) (20 mm diameter sphere). The PA signals within the FOV were collected at different time points (1 h, 4 h, 8 h, 24 h, 48 h), and the average intensities within the regions of interest (ROIs) were quantitatively analyzed using Osirix. Simultaneously, corresponding three dimensional images were reconstructed for a better observation of the structural details.

### NIR-II fluorescence imaging system

The emission light was passed through a 980 nm filter (Thorlabs, FEL1000) and a laser of power density at 40 mW cm^−2^ was used to enable optimal temporal resolution. The exposure time varied from 10 to 1000 ms or more depending on the brightness of the fluorescence and the speed of the camera. The images were acquired and analyzed by Light Field software. The further analyses of the fluorescence images were performed by Image J2x (Rawak Software Inc., Stuttgart, Germany) or Graphpad Prism 8 (GraphPad Software Inc., San Diego, CA, USA).

The fluorescence images of vessels were acquired on NIR-II fluorescence imaging setup previously described. Mice were placed in the supine position after the injection of SPN-PT (100 µL, 100 µg mL^−1^). FOVs were adjusted to cover the central vessels of left/right hind limb and fore limb. The NIR-II fluorescence imaging was performed immediately after the intravenous injection (i.v.) via tail vein. The exposure time was set to 100 ms.

For in vivo imaging of tumors, 143B tumor bearing mice were intravenously injected with either SPN-PT (100 µL, 100 µg mL^−1^) or SPN-SP (100 µL, 100 µg mL^−1^), and anesthetized with 2% isoflurane in oxygen and placed with supine position. The NIR-II fluorescence intensities within the tumor region were monitored following the injection (1 h, 4 h, 8 h, 24 h, 48 h) to compare the targeting ability of SPN-PT with SPN-SP. The exposure time was set to 200 ms.

In addition, the biodistribution of SPN-PT in 143B tumor-bearing mice were measured through ex vivo NIR-II fluorescence imaging of major organs (heart, liver, spleen, lung, kidney, brain, stomach, intestine, skin, muscle) and tumors from mice sacrificed on 48 h post injection of SPN-PT.

### Half-life of SPN-PT

To determine the concentration of SPN-PT in serum, different concentrations of SPN-PT (0, 2, 5, 8, 10, 12, 20 µg mL^−1^) were collected by heparinized capillary tubes and NIR-II fluorescent signals (excited at 808 nm, 980 nm filter, 20 ms exposure) were collected to determine the standard curve. Blood was firstly sampled as a reference before injection, using the same heparinized capillary tubes. Then Blood samples were collected from the end of the tail of the mice administrated with SPN-PT (intravenous injection, 100 µL, 100 µg mL^−1^) at 5 min, 15 min, 30 min, 45 min, 1 h, 2 h, 4 h, 6.5 h, 8 h, 12 h, 24 h post injection. The collected blood samples were also imaged by NIR-II fluorescence imaging system (808 nm, 980 nm filter, 20 ms’ exposure). SPN-PT in the blood was determined according to the standard curve aforementioned. According to Eq. (3), half-life was computed as t_1/2_ = ln (2) / K:

3 $$Y= (Y_{0} - Plateau)*exp(-K*X) + Plateau.$$

### Therapeutic efficacy in vivo

For an overall observation of the PTT capability driven by SPN-PT in 143B xenograft bearing mice, 100 µL of SPN-PT (100 µg mL^−1^), SPN-SP (100 µg mL^−1^) or PBS were systemically administrated into mice followed by PTT therapy (635 nm, 0.75 W cm^−2^, 6 min) and heating curves were acquired using an IR thermal camera. Two days (48 h) after the PTT therapy, the tumors and main organs were excised and placed in the NIR-II fluorescence device. Images were captured with 980 nm filter under the 808 nm laser excitation to see the biodistribution of SPN-PT in mice. It was found that the strong NIR-II fluorescence signal was mainly observed in the tumor, liver and spleen.

To specifically estimate therapeutic efficiency of SPN-PT in vivo, tumor-bearing mice (20 in total, of similar tumor volumes) were randomly divided into four groups on 14 d after inoculation, namely PBS group (systemic administrated with 100 µL of PBS), SPN-PT group (intravenous injection of 100 µL of 100 µg mL^−1^ SPN-PT), PBS+laser group (100 µL PBS followed by laser irradiation 24 h post injection, 635 nm, 0.75 W cm^−2^), SPN-PT+laser group (100 µL SPN-PT injected via tail vein and laser irradiation performed 24 h post injection, 635 nm, 0.75 W cm^−2^). Those mice who received laser irradiation were anesthetized with isoflurane in advance, and were exposed to 635 nm laser at the tumor region for 10 min with a power density of 0.75 W cm^−2^. Afterwards, these mice were housed under SPF conditions. Tumor size and body weight of mice were monitored and recorded every other day until they were sacrificed on 15 d post treatments.

To investigate the therapeutic efficacy on histological level, mice were sacrificed on 24 h after different treatments. Immediately after the sacrifice, the tumors tissues were resected meticulously, thus fixed in 4% neutral-buffered paraformaldehyde and embedded in paraffin for H&E staining.

### Generation of singlet oxygen in vivo

To evaluate the PDT effects in vivo, the generation of singlet oxygen were detected using SOSG probe (Invitrogen). The tumor bearing mice was intravenously injected with either SPN-PT (100 µg mL^−1^, 100 µL) or SPN-SP (100 µg mL^−1^, 100 µL) and treated with or without laser irradiation 24 h post the injection. SOSG (50 µM, 30 µL) probe was intratumorally injected into the tumor 30 min before the treatment. Then the region of tumor was irradiated with 635 nm laser (0.75 W cm^−2^) for 10 min at a discontinuous manner to maintain a low temperature below 43 ºC. The mice were then euthanized and the tumors were excised and fixed in 4% paraformaldehyde for histological analysis. The in vivo ^1^O_2_ generation was evaluated by comparing fluorescence intensity of SOSG using CLSM imaging. The cell nucleus was stained with DAPI showing blue fluorescence signal and green fluorescence showed the signals from SOSG (*Ex*/*Em* = 504/525 nm).

### Hematological analysis

To evaluate the biocompatibility of SPN-PT-based therapies in vivo, blood samples were collected at the endpoint of the therapies from the mice in different groups (PBS, SPN-PT, PBS+laser, SPN-PT+laser) and were administered to blood chemistry profile analyses, including alanine aminotransferase (ALT), aspartate aminotransferase (AST), urea (URE), and creatinine (Cre).

### Statistical analysis

Statistical comparisons among different groups at different time points were performed using two-way analysis of variance (Two-way ANOVA). Differences were considered as statistically significant at the level of *p* < 0.05 (*p* < 0.05, *; *p* < 0.01, **; *p* < 0.001, ***; *p* < 0.0001, ****). All data were shown as mean ± standard deviation (SD), n ≥ 3.

## Supplementary Information

**Additional file 1.** Supporting information of Figure S1–S13.

**Additional file 2: Movie S1.** Dynamic viewing of the 3D structure by PA imaging for 143B tumor in mice injected with SPN-PT at 1 h.

**Additional file 3: Movie S2.** Dynamic viewing of the 3D structure by PA imaging for 143B tumor in mice injected with SPN-PT at 4 h.

**Additional file 4: Movie S3.** Dynamic viewing of the 3D structure by PA imaging for 143B tumor in mice injected with SPN-PT at 8 h.

**Additional file 5: Movie S4.** Dynamic viewing of the 3D structure by PA imaging for 143B tumor in mice injected with SPN-PT at 24 h.
