# Supplementary material for: Peptide-based semiconducting polymer nanoparticles for osteosarcoma-targeted NIR-II fluorescence/NIR-I photoacoustic dual-model imaging and photothermal/photodynamic therapies
Source: J Nanobiotechnology. 2022 Jan 21;20:44. doi: 10.1186/s12951-022-01249-4 (PMC8780402; doi:10.1186/s12951-022-01249-4)
Supplement: Supplementary file 1 — Additional file 1. Supporting information of Figure S1–S13. [file 12951_2022_1249_MOESM1_ESM.docx]

**Additional file 1 for:**

Peptide-Based Semiconducting Polymer Nanoparticles for Osteosarcoma-Targeted NIR-II Fluorescence/NIR-I Photoacoustic Dual-model Imaging and Photothermal/Photodynamic Therapies


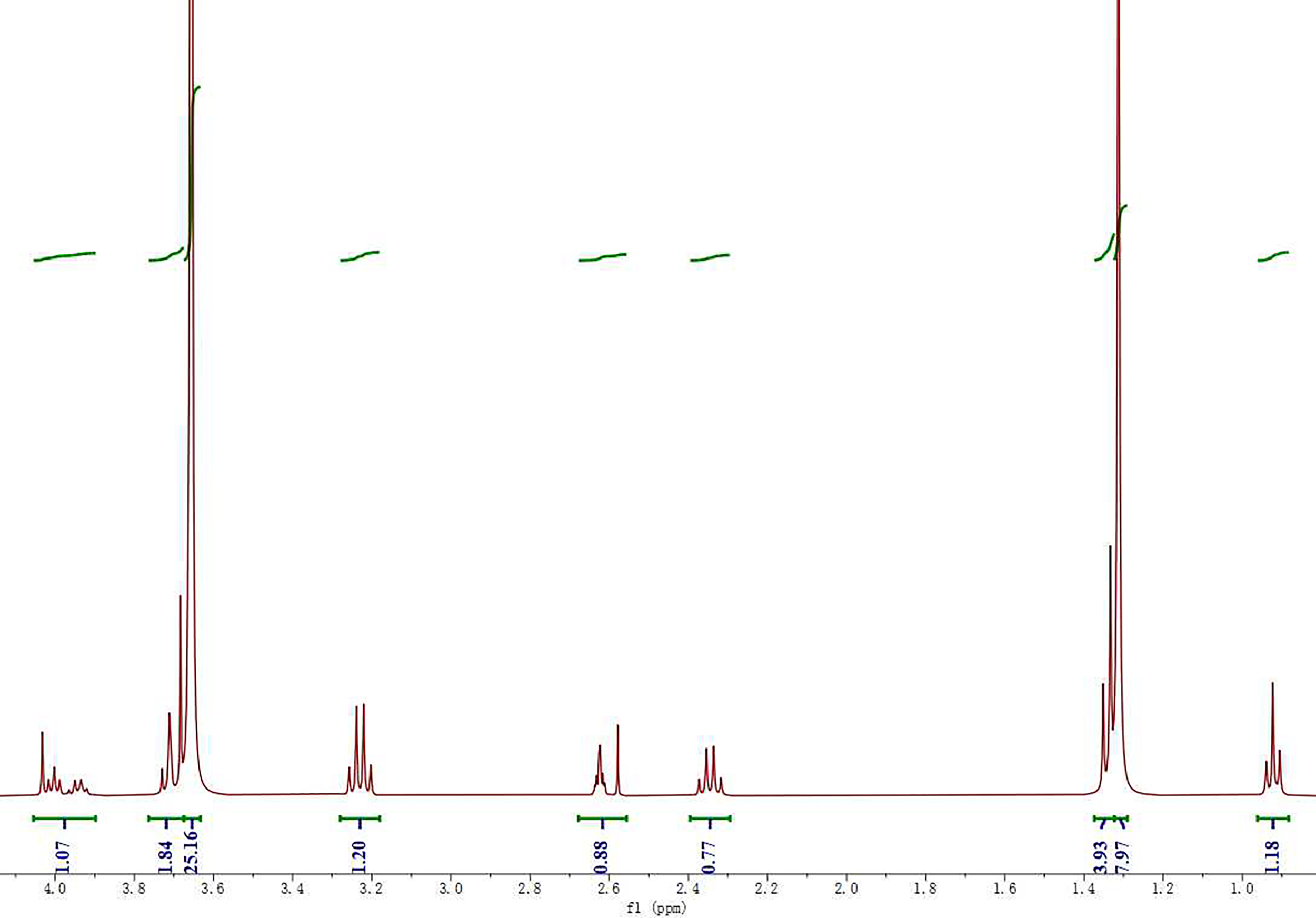


**Figure S1.** ^1^H NMR spectrum of DSPE-PEG_2000_-COOH in CD_3_OD.


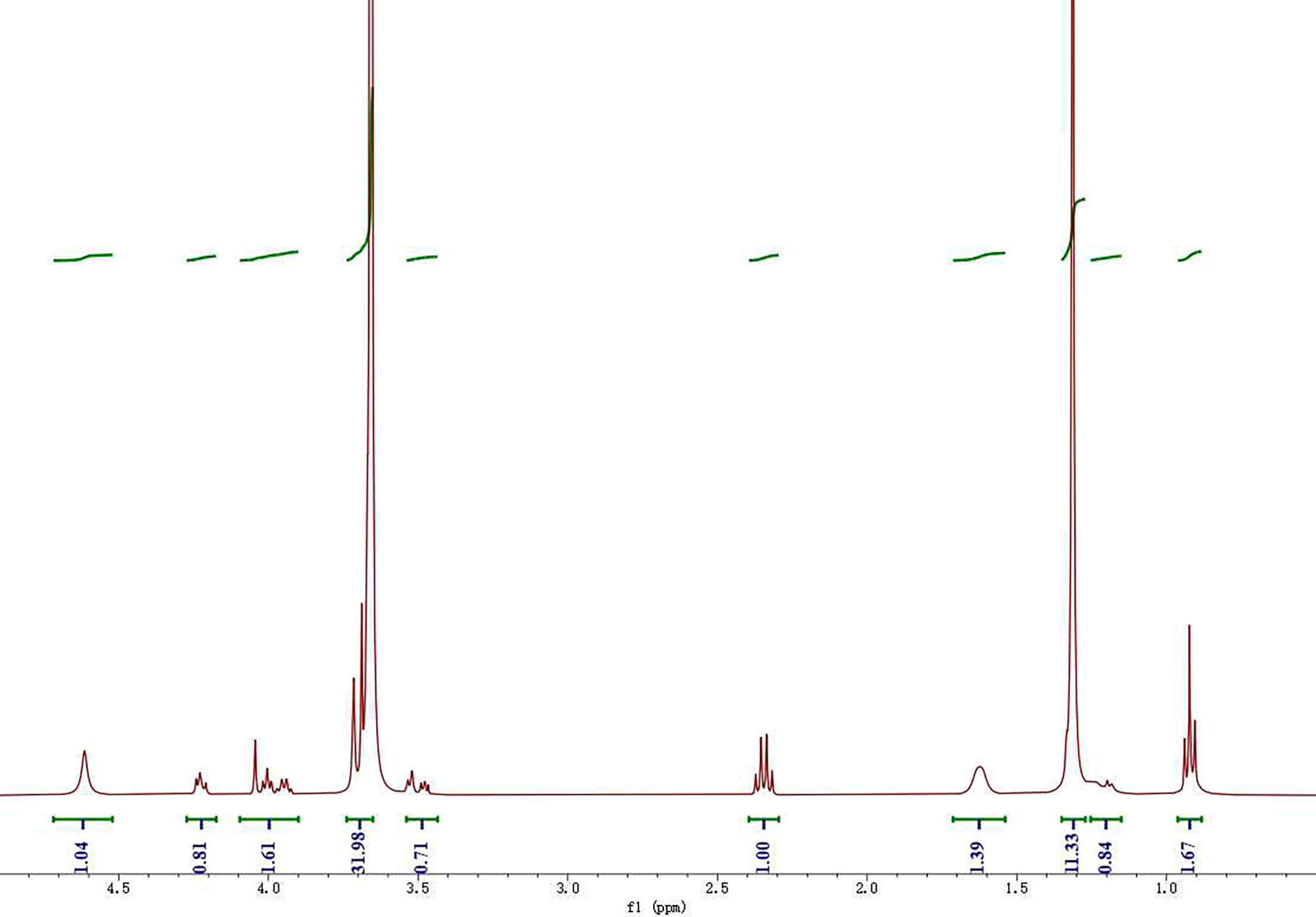


**Figure S2.** ^1^H NMR spectrum of PEG-PT in CD_3_OD.


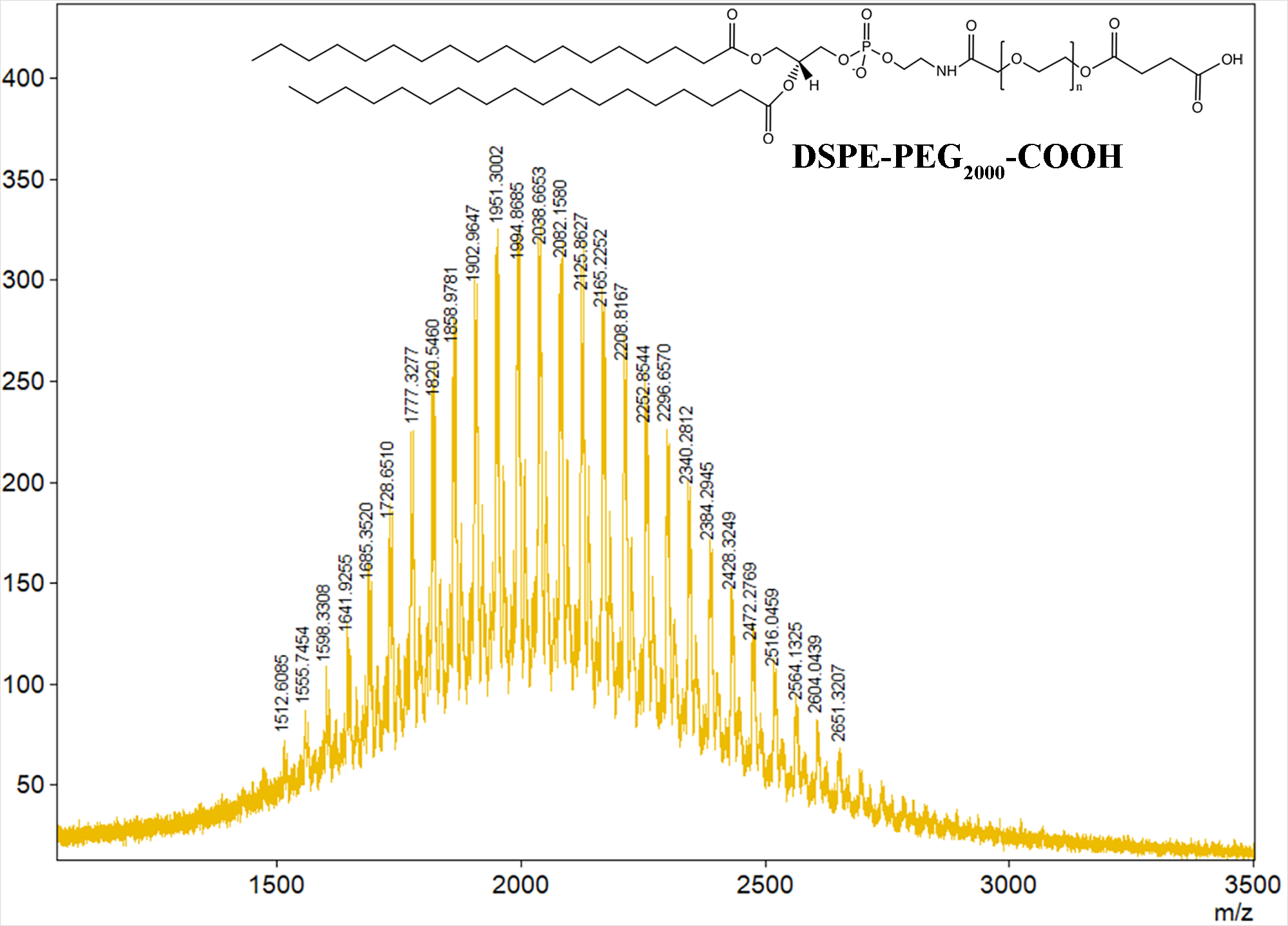


**Figure S3.** MALDI-TOF mass spectrum of DSPE-PEG_2000_-COOH in HCCA.


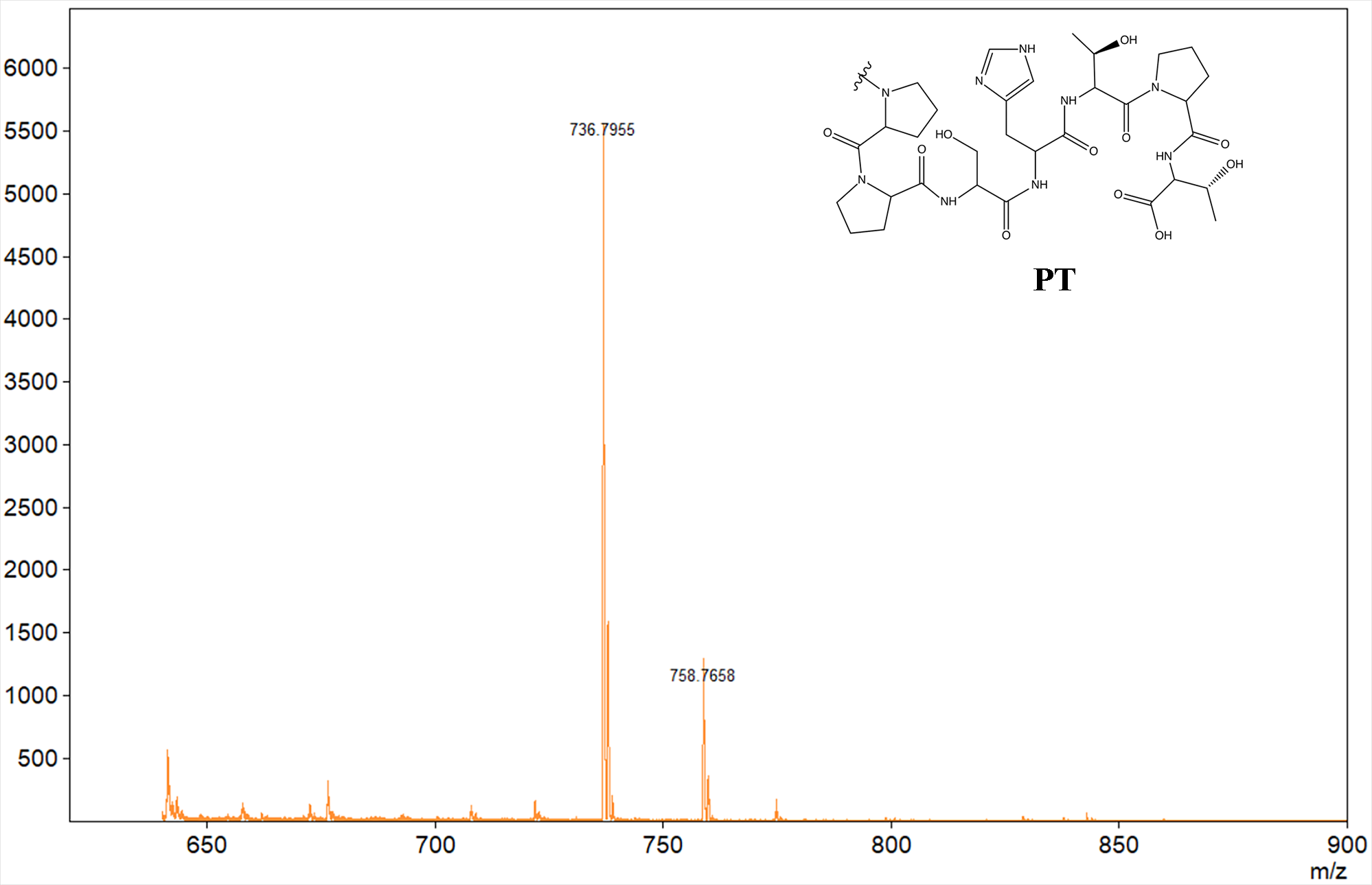


**Figure S4.** MALDI-TOF mass spectrum of peptide PT in HCCA.


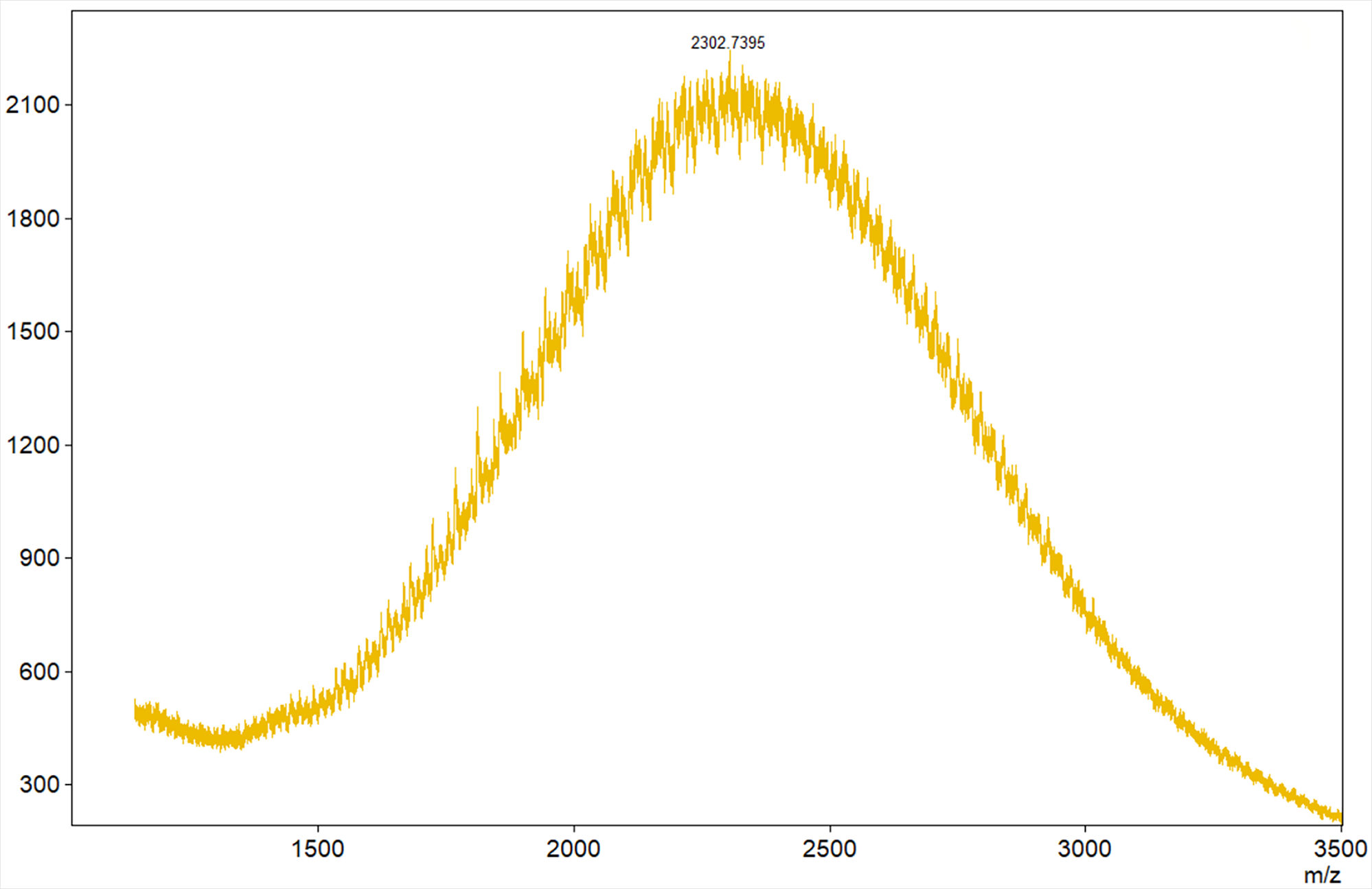


**Figure S5.** MALDI-TOF mass spectrum of PEG-PT IN HCCA.


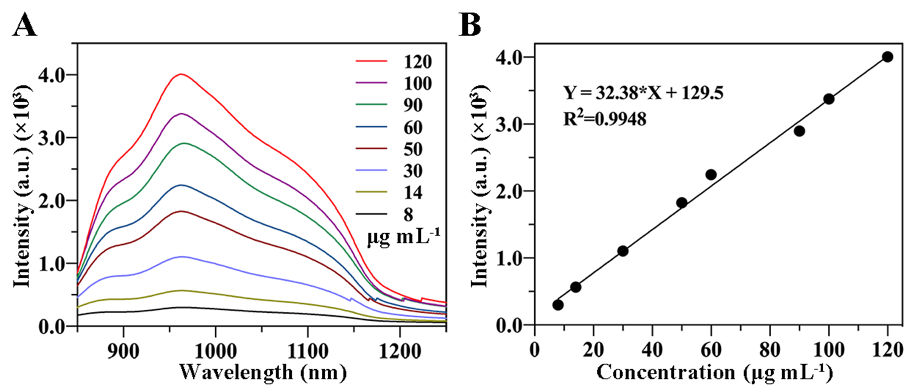


**Figure S6.** A) Fluorescence emission spectra of SPN-PT at different concentrations. B) The emission standard curve of SPN-PT at 961 nm.


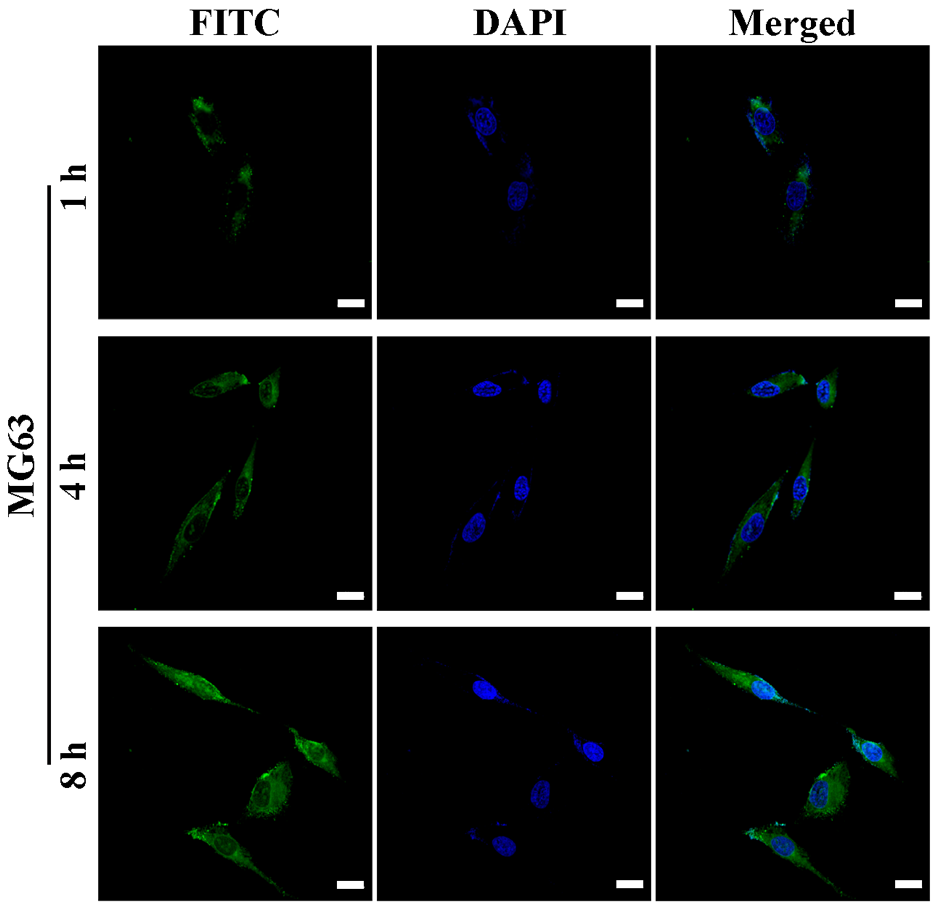


**Figure S7.** Cellular uptake of FITC-PT into MG63 cells. MG63 cells were incubated with FITC-PT (20 μg mL^-1^) for 1 h, 4 h, 8 h before CLSM imaging. Scale bar = 20 μm.


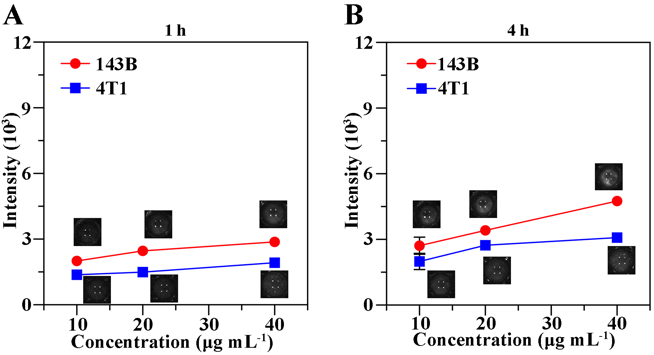


**Figure S8.** NIR-II fluorescence imaging of 143B cells incubated with different concentrations of SPN-PT (from left to right: 10 μg mL^-1^, 20 μg mL^-1^, 40 μg mL^-1^) for 1 hour (A) and 4 hours (B).


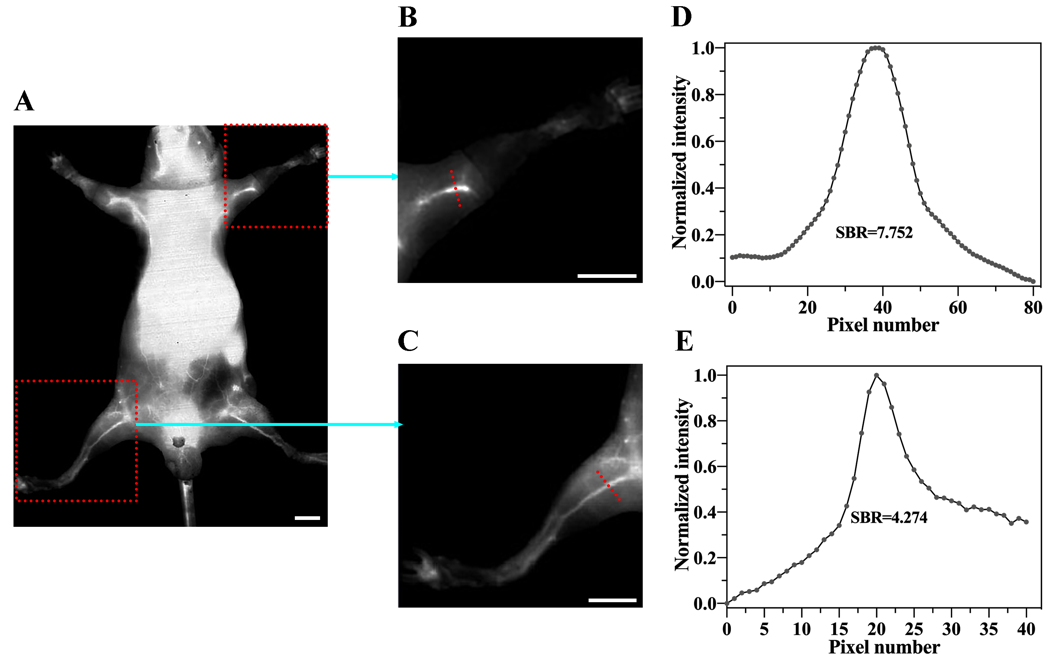


**Figure S9.** The NIR-II fluorescence imaging for vascular of body (A), fore limb (B) and hind limb (C). Scale bar = 5 mm. (D) and (E) were corresponding normalized fluorescence intensities of cross-section profiles along the red dotted lines in (B) and (C).


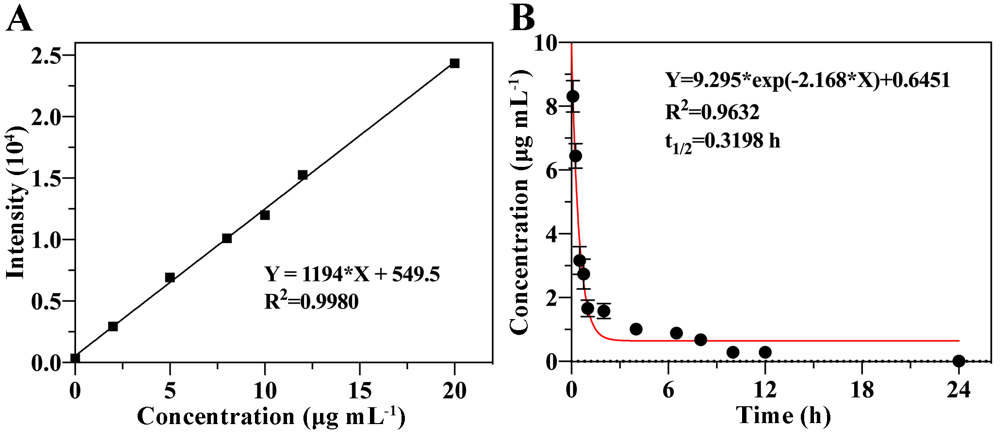


**Figure S10.** Calculation of half-life of SPN-PT in vivo. A) The standard curve was collected using heparinized capillary tubes. B) The serum concentration of SPN-PT was determined by NIR-II fluorescence intensity of serum collected at different time after systemic administration of SPN-PT (100 µg mL^-1^, 100 µL), in accordance to the standard curve in (A). Data were shown as mean ± SD, n = 3.


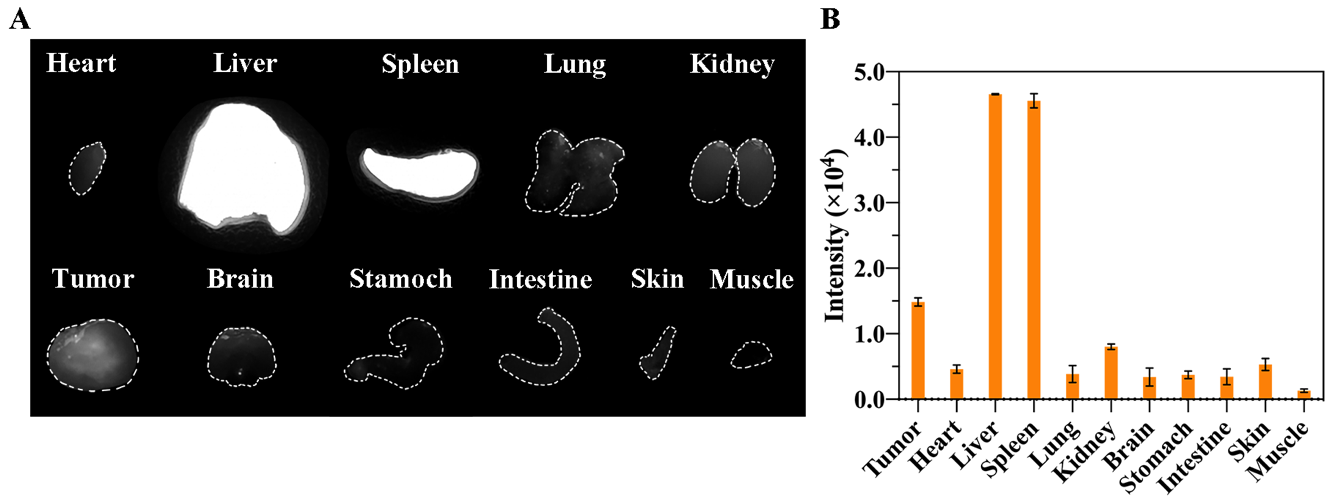


**Figure S11.** Representative NIR-II fluorescence images (A) and corresponding quantitation of NIR-II fluorescence intensities (B) in the region of major organs and tumors. The fluorescence was mainly observed in the tumor, liver and spleen. Data were shown as mean ± SD, n = 3.


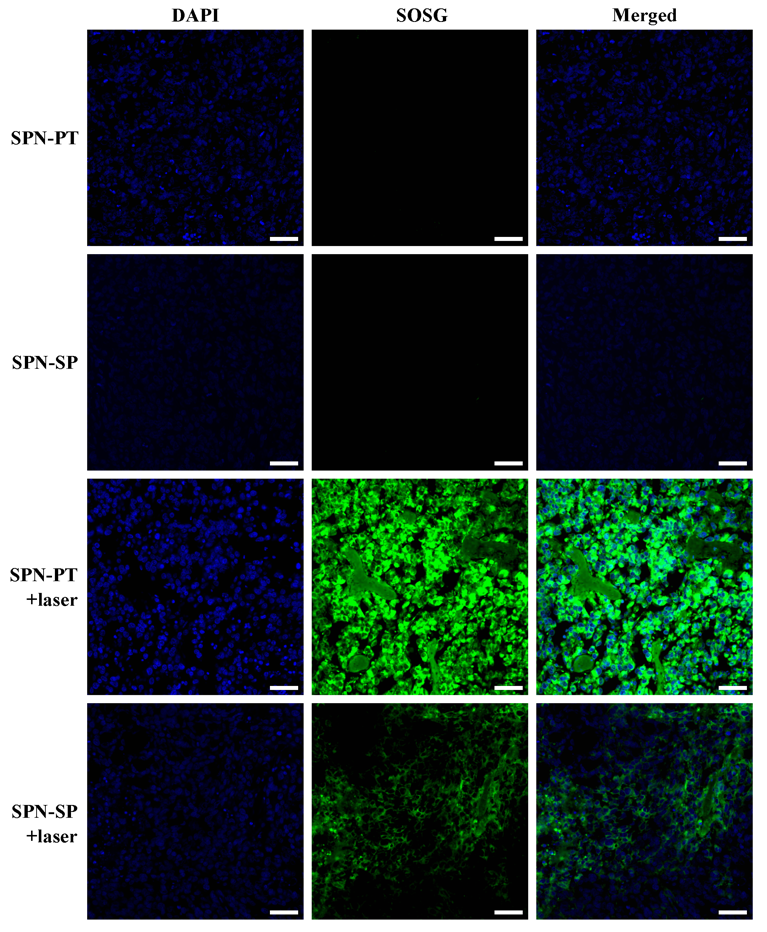


**Figure S12.** Confocal fluorescence images of tumor tissues from the SPN-PT or SPN-SP treated ((100 µg mL^-1^, 100 µL) mice with or without laser irradiation (635 nm, 0.75 W cm^−2^) for 10 min. Scale bar = 50 µm.


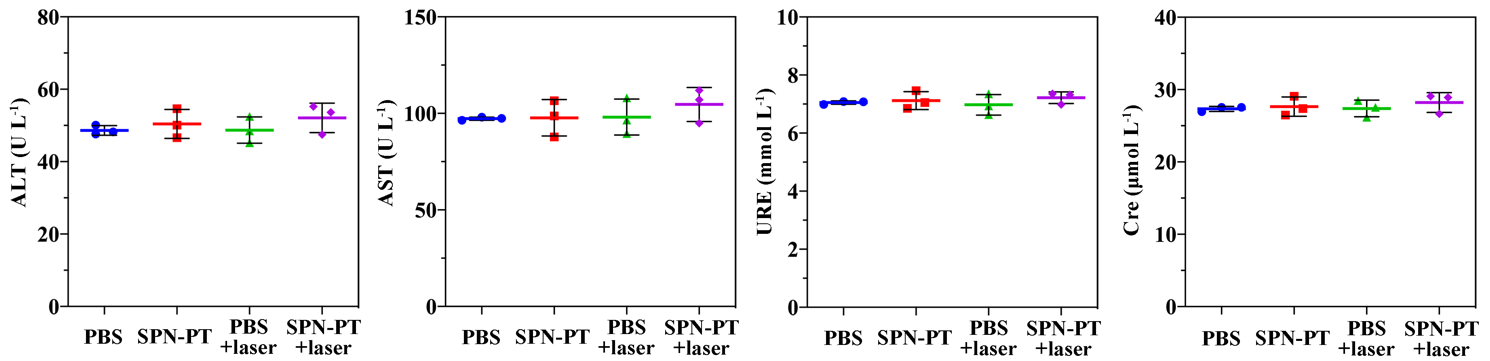


**Figure S13.** Blood chemistry profile analyses, including alanine aminotransferase (ALT), aspartate

aminotransferase (AST), urea (URE), and creatinine (Cre), for mice with different treatments at the therapeutic endpoint. Data were shown as mean ± SD, n = 3.
